# *polyamine uptake transporter 2* (*put2*) and decaying seeds enhance phyA-mediated germination by overcoming PIF1 repression of germination

**DOI:** 10.1371/journal.pgen.1008292

**Published:** 2019-07-24

**Authors:** Woohyun Kim, Sanja Ćavar Zeljković, Urszula Piskurewicz, Christian Megies, Petr Tarkowski, Luis Lopez-Molina

**Affiliations:** 1 Department of Botany and Plant Biology, University of Geneva, Geneva, Switzerland; 2 Institute of Genetics and Genomics in Geneva (iGE3), University of Geneva, Geneva, Switzerland; 3 Central Laboratories and Research Support, Faculty of Science, Palacký University, Olomouc, Czech Republic; 4 Department of Genetic Resources for Vegetables, Medicinal and Special Plants, Crop Research Institute, Olomouc, Czech Republic; Wake Forest University, UNITED STATES

## Abstract

Red light promotes germination after activating phytochrome phyB, which destabilizes the germination repressor PIF1. Early upon seed imbibition, canopy light, unfavorable for photosynthesis, represses germination by stabilizing PIF1 after inactivating phyB. Paradoxically, later upon imbibition, canopy light stimulates germination after activating phytochrome phyA. phyA-mediated germination is poorly understood and, intriguingly, is inefficient, compared to phyB-mediated germination, raising the question of its physiological significance. A genetic screen identified *polyamine uptake transporter 2* (*put2*) mutants that overaccumulate polyamines, a class of antioxidant polycations implicated in numerous cellular functions, which we found promote phyA-mediated germination. In WT seeds, our data suggest that canopy light represses polyamines accumulation through PIF1 while red light promotes polyamines accumulation. We show that canopy light also downregulates PIF1 levels, through phyA; however, PIF1 reaccumulates rapidly, which limits phyA-mediated germination. High polyamines levels in decaying seeds bypass PIF1 repression of germination and stimulate phyA-mediated germination, suggesting an adaptive mechanism promoting survival when viability is compromised.

## Author summary

Canopy light, unfavorable for photosynthesis, elicits paradoxical responses in seeds. Depending on the timing of irradiation upon seed imbibition, canopy light can either block or promote germination. The promotion effect is mediated by the light sensor phyA and, intriguingly, it is poorly efficient when compared to that of red light, which is favorable for photosynthesis. Why would germination be stimulated by canopy light, which is not favorable for young seedling photosynthesis? We show that phyA-mediated germination is greatly enhanced in decaying seeds, which overaccumulate polyamines, a class of antioxidant molecules that promote germination. We propose an adaptive rationale for phyA-mediated germination: in decaying seeds it becomes advantageous to germinate even under canopy light as a last chance for seedling survival.

## Introduction

Seeds are capsules maintaining the plant embryo in a resistant state and promoting plant dispersal. To successfully produce a seedling, seeds are able to remain viable over time and block their germination under potentially fatal conditions for the seedling.

However, over time seeds irremediably accumulate oxidative events, which eventually will compromise their viability [1,2]. Seeds limit oxidative damage through antioxidants or physical barriers limiting oxygen access to the embryo [3–5]. Polyamines (PAs) are small polycations ubiquitously present in all living organisms where they regulate numerous fundamental cellular processes, including DNA replication, transcription, translation and post-translational modification, cell proliferation, cell cycle regulation and programmed cell death [6–8]. However, how PAs perform these functions is poorly understood. In response to oxidative stress PA levels rise and protect cells by scavenging reactive oxygen species (ROS) and by increasing antioxidant enzymes activity in leaves [9–13]. However, whether PAs accumulate in Arabidopsis seeds after oxidative stress is unknown.

Arabidopsis seeds control their germination after perceiving abiotic parameters in their environment. This triggers signaling responses leading to opposite level changes of abscisic acid (ABA) and gibberellic acid (GA), two hormones that repress and promote germination, respectively [2].

The quality of light perceived by the light receptor phytochromes phyB and phyA exerts a profound influence on seed germination. Phytochromes control the abundance of the transcription factor PHYTOCHROME-INTERACTING FACTOR 1 (PIF1), a key germination repressor regulating ABA and GA levels in seeds [14]. Red (R) light, favorable for photosynthesis, promotes germination while canopy light, enriched in far red (FR) light, represses germination. Early upon seed imbibition, a R pulse photoconverts phyB into its active P_fr_B form that interacts with PIF1 and promotes its destruction, thus promoting germination. In contrast, a pulse of FR light photoconverts phyB into its inactive P_r_B form, which leads to PIF1 stabilization, thus preventing germination [15].

Paradoxically, a second FR light pulse applied later on upon imbibition promotes germination by activating phyA [16–18]. Intriguingly, unlike R light, FR light promotes germination inefficiently and erratically among seed batches. This low efficiency was linked to limiting phyA levels and strong ABA-dependent responses early upon seed imbibition [16,17]. However, regulation of endogenous PIF1 levels by FR light through phyA has not been reported. phyA-mediated germination was interpreted as a last chance to form a seedling despite the presence of unfavorable canopy light [17,19]. Yet, its low efficiency suggests that its physiological significance is not fully understood.

Here we found that recessive mutations in *POLYAMINE UPTAKE TRANSPORTER 2* (*PUT2*) enhance phyA-mediated germination. *put2* seeds overaccumulate PAs and we show that PAs stimulate phyA-mediated germination. In WT seeds, our data suggest that upon phyB inactivation after an early FR pulse, PIF1 represses PAs accumulation. phyB activation by R light promotes PIF1 downregulation and PAs accumulation. Upon phyA activation by a second FR pulse, PIF1 is downregulated but, surprisingly, increase in PAs accumulation does not take place. We propose that this differential regulation of PAs levels arises from the duration of PIF1 extinction time, which is longer after R than after FR light irradiation. Accelerated aging procedures stimulated PAs accumulation and markedly enhanced phyA-mediated germination without downregulating PIF1 levels. Our results suggest that decaying seeds bypass PIF1-dependent repression of PAs accumulation to enhance phyA-mediated germination even under unfavorable canopy light conditions.

## Results

**Fig 1:**
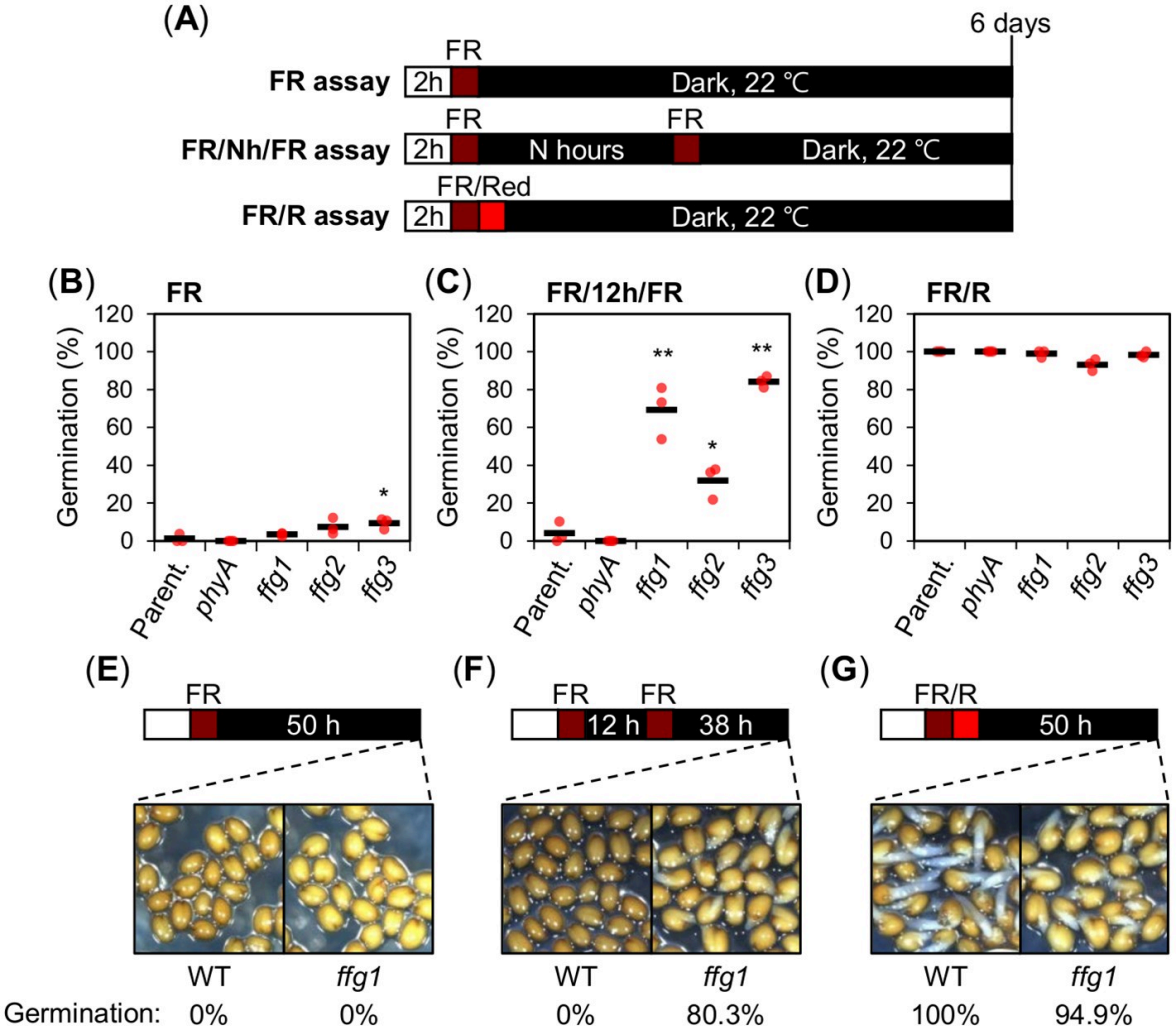
Identification of *ffg* mutants. (A) Diagram depicts the different light treatment assays applied to seeds and used throughout this study. In all assays seeds were kept in darkness at 22°C following light treatments and germination percentages were determined as radicle protrusion after 6 days. “FR assay”: a FR pulse (3.69 μmol m^-2^ s^-1^) is applied for 5 minutes (min) on seeds previously imbibed under constant white light for 2 hours (h); “FR/Nh/FR assay”: same as a FR assay but an additional FR pulse (3.69 μmol m^-2^ s^-1^) is applied for 5 min on seeds N h after the first one. “FR/R assay”: same as the FR assay where the FR pulse is immediately followed by a R pulse (14.92 μmol m^-2^ s^-1^) applied for 5 min. (B-D) Scatter dot plots show germination percentages of parental line (Parent.: WT(Col-0)/*pGA3ox1*::*LUC*), *phyA-211* (*phyA*) and *ffg1*—*ffg3* mutant seeds in a FR (B), FR/12h/FR (C) and FR/R assay (D). One biological seed batch sample was used to measure the average seed germination percentage using three technical repetitions. For each repetition, the germination percentage of 50–65 seeds is shown by a red dot. The average germination percentage for the three repetitions is represented by a horizontal black bar. One-way ANOVA followed by a Tukey HSD test shows statistically different values compared to Parent. (* p<0.05 and ** p<0.01). (E-G) Representative pictures of WT and *ffg1* seeds exposed to a FR (E), FR/12h/FR (F) and FR/R assay (G). Pictures were taken at the time shown in the diagram. Germination percentages are shown under each picture.

Hereafter a “FR assay” refers to the procedure where seeds are irradiated with a single far red (FR) pulse early upon imbibition (Fig 1A). In a “FR/Nh/FR assay” seeds are irradiated with two FR pulses separated by an N hour (h) time interval, typically 48 hours (N = 48) (Fig 1A). In a “FR/R assay” seeds are irradiated with a FR pulse early upon imbibition immediately followed by a red (R) light pulse (Fig 1A; see Materials and methods).

### Identification of *POLYAMINE UPTAKE TRANSPORTER 2* (*PUT2*) in a genetic screen for mutants with enhanced phyA-mediated germination responses

To better understand why phyA-mediated germination is limited in seeds, we sought to identify negative regulators of phyA signaling by screening for mutants displaying enhanced phyA-dependent seed germination responses. We generated a transgenic line carrying a firefly *LUCIFERASE* (*LUC*) reporter gene under the control of *GA3ox1* promoter sequences (*pGA3ox1*::*LUC*) [20]. High *GA3ox1* expression is characteristic of seeds undergoing germination [21]. *pGA3ox1*::*LUC* seeds were mutagenized using ethyl methanesulfonate (EMS).

FR/12h/FR assays lead to lower phyA-dependent germination relative to FR/48h/FR assays [16,17]. Thus, to identify negative regulators of phyA-dependent germination, we screened for *pGA3ox1*::*LUC* mutant seeds displaying high LUC bioluminescence and germination in a FR/12h/FR assay (S1A Fig; see Materials and methods for details). Mutants identified in this manner were propagated and the resulting seed progeny was further studied. This led to identify 5 recessive and independent mutants, named *fr/fr germination 1*–*5* (*ffg1*-*ffg5*) having enhanced germination in a FR/12h/FR assay relative to the parental non-mutagenized *pGA3ox1*::*LUC* (Parent.) line (Fig 1B–1D and S1B–S1D Fig).

Unsurprisingly, among these mutants, we found two mutants (*ffg4* and *ffg5*) having high germination percentage in a FR assay (S1B Fig). *ffg4* had a G-to-A transition at nucleotide 370 in *PIF1*/*PIL5* (At2g20180), which resulted in the substitution of Trp-94 with a Stop codon (S1E Fig). *ffg5* had a mutation at the splicing site of the third intron of the ABA biosynthetic gene *ABA1* (At5g67030) (S1F Fig). *PIF1*/*PIL5* and ABA were previously shown to negatively regulate phyB signaling and were therefore not further studied [17,22,23].

We also identified three independent recessive mutants that did not germinate in a FR assay but showed enhanced phyA-mediated germination (Fig 1B–1D). We selected *ffg1* for further study (Fig 1E–1G).

**Fig 2:**
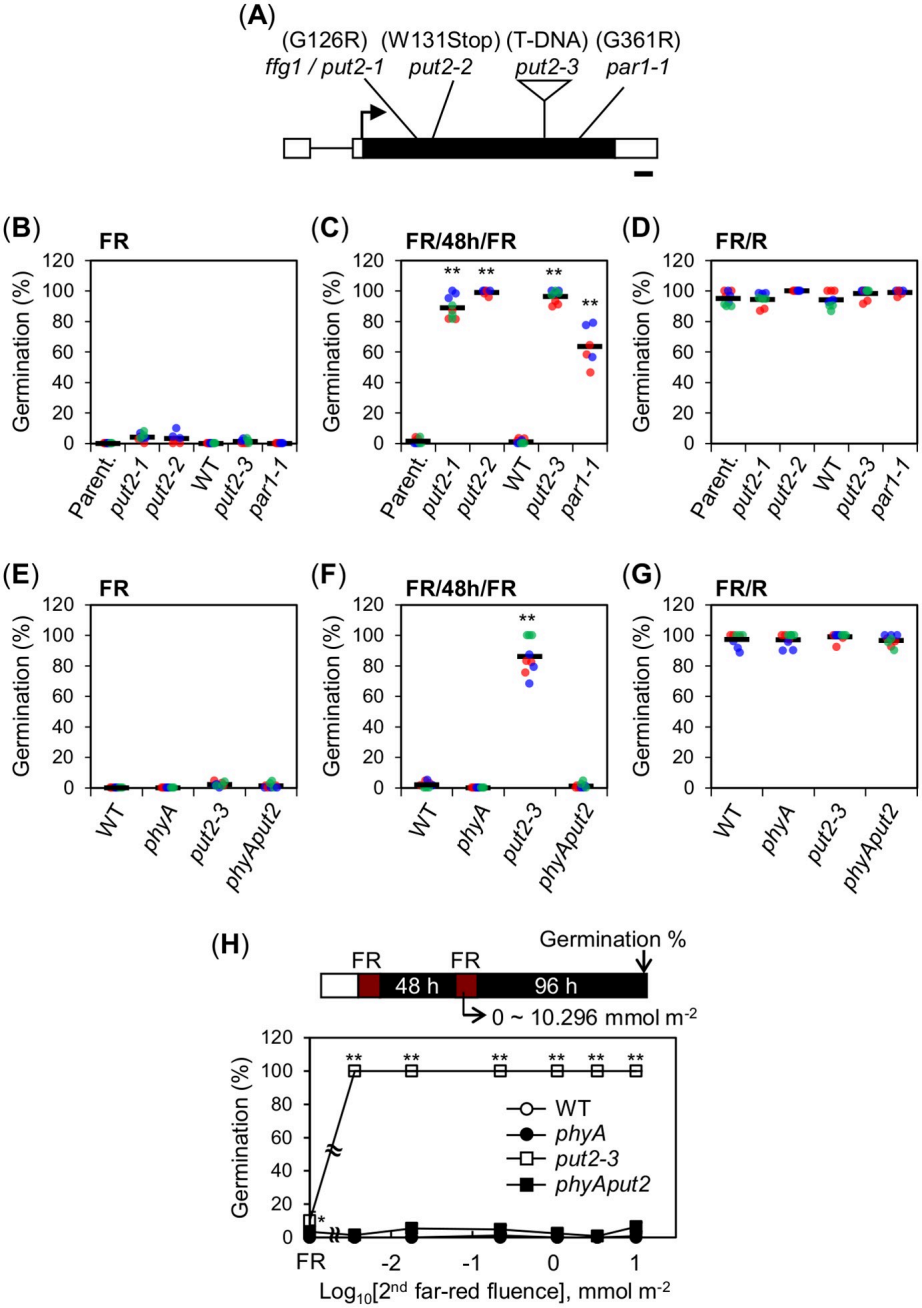
*PUT2* encodes a negative regulator of phyA signaling in seeds. (A) Diagram shows the genomic structure of the *PUT2* gene and the different mutant alleles used in this study. Black box: exon, black line: intron, white boxes: UTRs and arrow: transcription start site. Bar: 100 bp. (B-D) Scatter dot plots show germination percentages of 18-month-old wild type (WT) and different *put2* mutant seeds, alleles as indicated, exposed to a FR (B), FR/48h/FR (C) or FR/R assay (D). (E-G) Germination percentages of Col-0 (WT), *phyA-211* (*phyA*), *put2-3* and *phyA-211put2-3* (*phyAput2*) seeds exposed to a FR (E), FR/48h/FR (F) or FR/R assay (G). (H) Graph shows germination percentages of WT, *phyA*, *put2-3* and *phyAput2* seeds exposed to a FR/48h/FR assay using different FR light fluences for the second FR pulse (0 to 10.296 mmol m^-2^). FR indicates FR assay (No second FR pulse). For (B-G) two or three independent biological replicates, depending on the genotype used, were used and are represented with an individual color each (red, blue or green). For each biological replicate, three technical repetitions of the germination percentage of 50–65 seeds are shown by a dot (red, blue or green). The average germination percentage for all the technical repetitions is represented by a horizontal black bar. For (H) three technical repetitions (n = 50–65 each) were used for SD. Statistical treatment as in Fig 1B. Experiment in (H) was repeated with one biological seed batch sample, which provided similar results.

The *ffg1* locus was mapped to a 200 kbp interval on chromosome 1 (11.3 ~ 11.5 Mbp.). Sequencing analysis revealed that *ffg1* mutants had a G-to-A substitution at nucleotide 376 in the Arabidopsis locus At1g31830 (*PUT2* / *LAT4* (*L-AMINO ACID TRANSPORTER 4*) / *PAR1* (*PARAQUAT RESISTANT 1*) / *PQR2* (*PARAQUAT-RESISTANT 2*)), which converts Gly-126 to Arg. This G126R transition is hereafter referred as a *put2-1* mutant allele (Fig 2A).

*PUT2* encodes an amino acid permease family protein and *put2* mutants were reported to display resistance to paraquat (PQ), a methyl viologen widely used as a herbicide [24,25]. We found that *put2-1* mutants were resistant to PQ, indicating that PUT2 activity is defective in *put2-1* mutants (S2A Fig). This suggested that PUT2 negatively regulates phyA-mediated germination. We observed enhanced phyA-mediated germination in two previously reported independent *put2* mutant alleles, *par1-1* [24] and *par1-5* [24] (hereafter *put2-3*), and in a new one, named *put2-2*, identified in this study after screening for PQ-insensitive mutant plants (Fig 2B–2D and S2A Fig; see Materials and methods).

Expectedly, *phyAput2* double mutant seeds did not germinate in a FR/48h/FR assay, showing that the high percentage germination of *put2-3* mutants in response to FR light is mediated by phyA (Fig 2E–2G and S2B Fig). We also assessed germination percentages using different FR light fluences for the second FR pulse in a FR/48h/FR assay. *put2-3* seed germination was enhanced relative to that of WT seeds under all FR fluences considered (Fig 2H).

Altogether, these results confirm that *PUT2* encodes a negative regulator of phyA-mediated seed germination. We next sought to understand how *PUT2* represses phyA-mediated responses in seeds.

### *put2* mutant seeds have high polyamine levels

**Fig 3:**
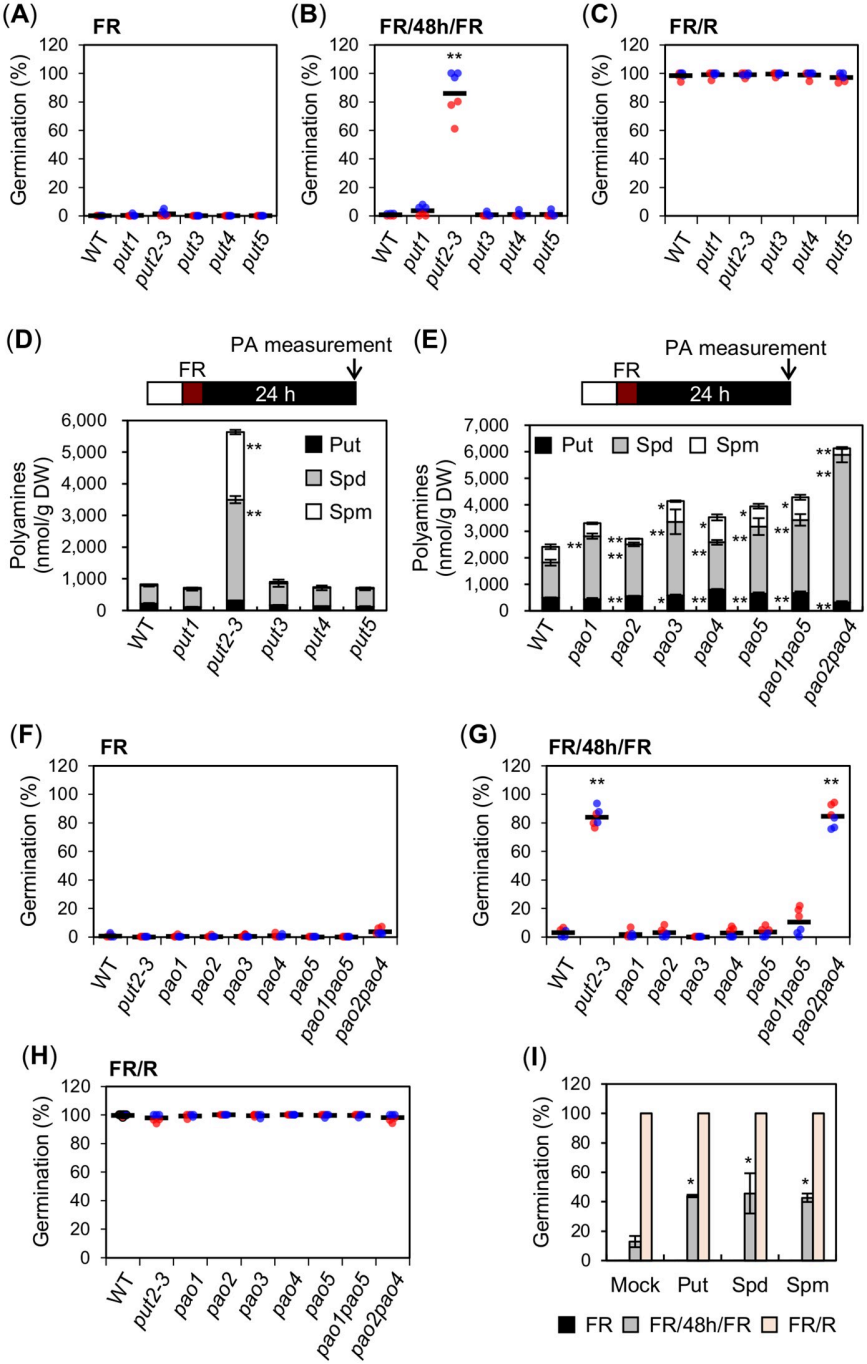
High PA levels are associated with enhanced phyA-mediated seed germination. (A-C) Scatter dot plots show germination percentages of WT and *put1*—*put5* seeds exposed to a FR (A), FR/48h/FR (B) or FR/R assay (C). (D) Free PA (Put, Spd and Spm) levels in WT and *put1*—*put5* seeds exposed to a FR assay. PAs were measured 24 h after the FR pulse. (E) Free PA (Put, Spd and Spm) levels in WT, *pao1*—*pao5*, *pao1pao5* and *pao2pao4* seeds exposed to a FR assay. PAs were measured 24 h after the FR pulse. (F-H) Germination percentages of WT, *put2-3*, *pao1*—*pao5*, *pao1pao5* and *pao2pao4* seeds exposed to FR (F), FR/48h/FR (G) or FR/R assay (H). (I) Germination percentage of WT seeds exposed to a FR, FR/48h/FR or FR/R assay in germination plates containing 100 μM of individual PAs as indicated. For the germination assays in (I), two independent seed batch samples (biological replicates) were used. Germination percentage was assessed once with 50–65 seeds for each biological replicate and percentage values were used to calculate average germination percentage and SD. For the germination assays in (A-C) and (F-H), two independent biological replicates were used and are represented with an individual color each (red or blue). For each biological replicate, three technical repetitions of the germination percentage of 50–65 seeds are shown by a colored dot (red or blue). The average germination percentage for all the technical repetitions is represented by a horizontal black bar. For (D and E) three technical repetitions were used for SD (n = 3). Statistical treatment as in Fig 1B.

PUT2 belongs to the amino acid/polyamine/organocation (APC) transporter superfamily, which has four homologs in Arabidopsis: PUT1, PUT3, PUT4 and PUT5 [26–28]. The highest homology is found with PUT1 (75% identities, 83% positives, 3% gaps), followed by PUT3 (67%, 82%, 3%), PUT5 (53%, 69%, 7%) and PUT4 (42%, 63%, 4%) (S3 Fig). Publicly available data show that *PUT2*, *PUT3* and *PUT4* are expressed in developing, mature and imbibed seeds; however, *PUT2* expression in developing seeds or upon seed imbibition is markedly higher relative to that of its homologs (S4 Fig). This suggested that *PUT2* is unique among its homologs in negatively regulating phyA-mediated seed germination. Indeed, the germination percentage of *put1*, *put3*, *put4* and *put5* mutant seeds exposed to a FR/48h/FR assay was low and similar to that of WT seeds (Fig 3A–3C and S5 Fig).

PUT2, PUT1 and PUT3 were shown to transport polyamines (PAs) in yeast and plants [27,29,30]. This suggested that enhanced phyA-mediated seed germination of *put2* seeds could result from defects in PAs cellular distribution or metabolism in seeds. The most abundant PAs in plants are putrescine (Put), spermidine (Spd) and spermine (Spm). Di-amine Put is made from arginine and is essential for plant survival [31]. Two spermidine synthases (SPDS1 and 2) are responsible for tri-amine Spd synthesis from Put, and spermine synthase (SPMS) is responsible for tetra-amine Spm synthesis from Spd. Spd, but not Spm, is essential for plant survival [32,33].

We measured free Put, Spd and Spm accumulation in seeds exposed to a FR assay 24h after FR light irradiation. In WT seeds, overall PAs levels, i.e. the sum of Put, Spd and Spm levels, were variable between seed batches ranging from about 600 nmol per gram of seed dry weight (nmol/g DW) to 1,800 nmol/g DW (S6A Fig). In every WT seed batch, Spd was the most abundant PA followed by Put and Spm. Overall PAs levels and the relative amounts of Put, Spd and Spm in *put1*, *put3*, *put4* and *put5* mutant seeds were similar to WT seeds (Fig 3D). In contrast, overall PAs levels in *put2-3* mutant seeds were systematically markedly higher relative to WT seeds among different seed batches, mostly due to higher Spd and Spm levels (Fig 3D and S6B Fig). In dry seeds, higher PAs levels were also specifically observed in *put2-3* mutants (S6C Fig). Similar results were obtained with *put2-1*, *put2-2* and *par1-1* alleles (S6D Fig).

These observations therefore suggest that high PAs levels could be responsible for the enhanced phyA-mediated seed germination in *put2* mutant seeds, i.e. that PAs are positive regulators of phyA signaling in seeds.

### High PA levels are associated with enhanced phyA-mediated seed germination

To further study the role of PAs in regulating phyA signaling in seeds, we considered assessing phyA-mediated responses in absence of endogenous PAs. However, PAs regulate fundamental cellular processes and mutations preventing PA biosynthesis in Arabidopsis are lethal, which makes this task challenging [8,31–33].

Instead, we considered identifying seeds with higher PAs levels. Five polyamine oxidases in Arabidopsis (PAO1-PAO5) catabolize PAs by converting Spm to Spd and Spd to Put [34]. Previous reports showed that *pao1*—*pao5* single mutants accumulate higher individual or overall PAs levels [35]. However, in some cases, as with *pao4* mutants, lower levels in individual PAs were reported [36]. PAs levels in *pao1*—*pao5* single mutant seeds were not previously reported. We observed moderately higher, i.e. less than twofold, overall PAs levels in *pao1*—*pao5* seeds relative to WT (Fig 3E).

PAO1 and PAO5 are localized in the cytosol whereas PAO2, PAO3 and PAO4 are localized in the peroxisome [34]. We measured PAs levels in *pao1pao5* and *pao2pao4* double mutants, defective in cytoplasmic and peroxisomal PAO activity, respectively [37]. We found more than twofold higher PAs levels in *pao2pao4* double mutants whereas *pao1pao5* double mutants had moderately higher, i.e. less than twofold, PAs levels (Fig 3E). Strikingly, only *pao2pao4* double mutant seed germination was markedly enhanced in a FR/48h/FR assay (Fig 3F–3H). Furthermore, we observed that addition of individual PAs in the germination plates enhanced phyA-mediated germination (Fig 3I).

Altogether, these observations support the hypothesis that PAs are positive regulators of phyA-mediated seed germination.

We sought to further evaluate this notion by identifying physiological conditions that could enhance PAs levels in seeds and whether they were associated with more efficient phyA-mediated germination.

### Artificial seed aging promotes PA accumulation and phyA-mediated seed germination

**Fig 4:**
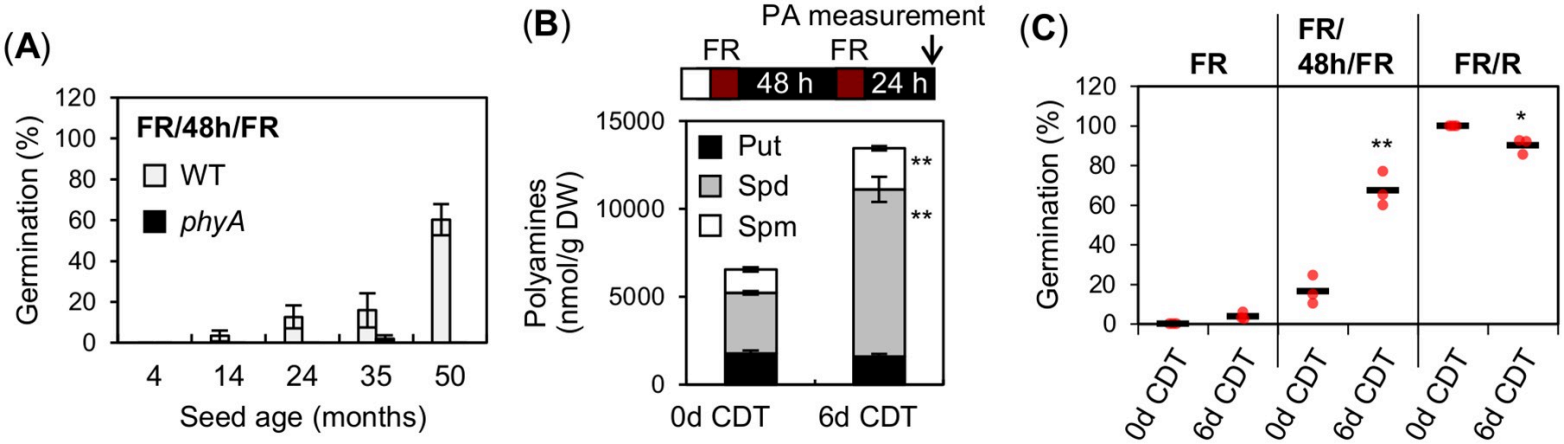
Seeds undergoing controlled deterioration treatment (CDT) increase PA levels and enhance phyA-mediated seed germination. (A) Histograms show germination percentages with WT and *phyA* seeds of different ages exposed to a FR/48h/FR assay. Three technical repetitions of the germination percentage of 50–65 seeds were used for SD. (B) Free PA (Put, Spd and Spm) levels in CDT-exposed WT seeds exposed to a FR/48h/FR assay. PA levels were measured 24 h after the second FR pulse. Three technical repetitions were used. (C) Germination percentages of WT seeds shown in (B) after exposure to a FR, FR/48h/FR or FR/R assay. For each repetition, the germination percentage of 50–65 seeds is shown by a red dot. The average germination percentage for the three repetitions is represented by a horizontal black bar. Statistical treatment as in Fig 1B. Experiments in (B) and (C) were repeated with two biological seed batch samples, which provided similar results (S7B, S8B and S8C Figs).

PAs act as antioxidants in plants where they accumulate in vegetative tissues [12,13] in response to oxidative stress. Seeds irremediably accumulate oxidative events as they age and oxidative stress is a major factor affecting seed viability [38–40]. Interestingly, we observed that the percentage of phyA-mediated seed germination markedly increased with old seed batches, reaching as much as 60% with five-year old seeds (Fig 4A). This experiment was performed with seeds produced at different times, which could lead to differences in germination among seed batches. Nevertheless, these observations are consistent with the notion that phyA-mediated germination increases with oxidative stress.

Whether oxidative stress promotes PAs accumulation in seeds was not previously investigated. To address this question, we subjected WT seeds to a controlled deterioration treatment (CDT), which promotes oxidative stress and artificially accelerates seed aging [4,40]. Increased oxidative stress upon seed exposure to CDT was verified by measuring superoxide O_2_^-^ levels in seeds (S7A Fig). Next, seeds that had undergone CDT were exposed to a FR/48h/FR assay and PAs levels were measured 24h after the second FR light pulse. As anticipated, PAs levels increased after exposure to 6 days of CDT (Fig 4B and S7B Fig). The increase in PAs levels was not as pronounced as in *put2* mutants (Fig 3D).

Consistent with previous results, seeds exposed to white light or a FR/R assay decreased their seed germination percentage from 100% to 90% after exposure to CDT, indicating that seeds that had been exposed to CDT started to lose their viability [4,41] (Fig 4C and S8A, S8B and S8C Fig). In contrast, the germination percentage in a FR/48h/FR assay markedly increased from 12% to 67% after exposure to CDT, i.e. nearer to the germination percentage observed in a FR/R assay (Fig 4C). A similar trend was obtained with independent seed batches exposed to CDT (S8B and S8C Fig).

Altogether, these data further strengthen the notion that high endogenous PAs levels in seeds enhance phyA-mediated germination.

We next sought to better understand 1) what makes phyA-mediated germination less efficient than phyB-mediated germination and 2) whether this low efficiency reflects how PA levels are regulated by light in seeds. These questions were addressed by studying the role of PIF1, a key light regulated germination repressor, in regulating endogenous PAs levels. We first monitored endogenous PIF1 accumulation in seeds exposed to light treatments conducive of phyA-mediated germination, which was not previously reported.

### Germination stimulated by FR and R light differs by the duration of PIF1 extinction time after FR and R light irradiation, respectively

**Fig 5:**
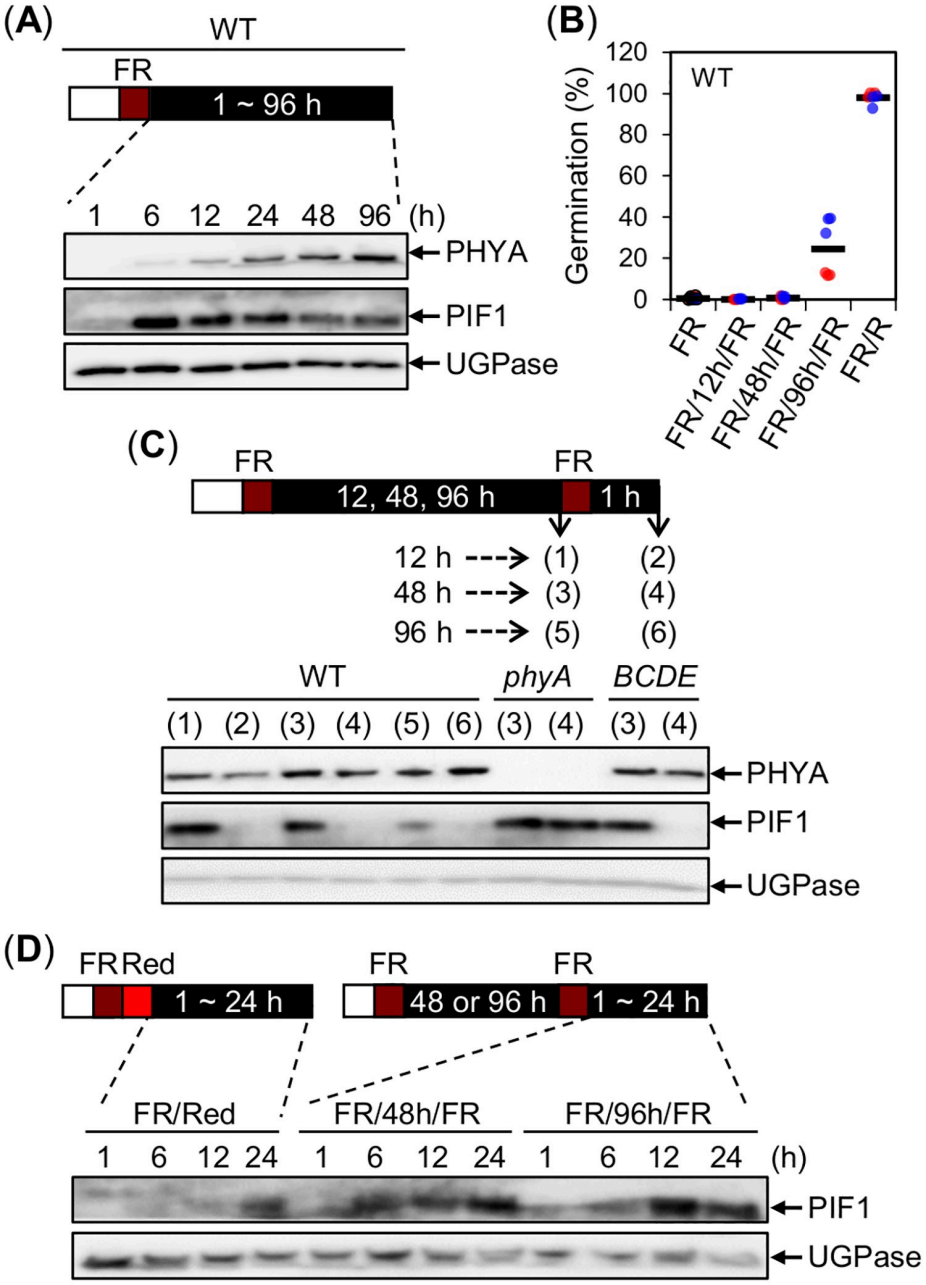
PIF1 extinction time is shorter after FR light irradiation than after R light irradiation. (A) Protein gel blot analysis of phyA and PIF1 protein levels in WT seeds exposed to a FR assay at the indicated time points after the FR light pulse. (B) Germination percentage of WT seeds exposed to a FR, FR/12h/FR, FR/48h/FR, FR/96h/FR or FR/R assay. Two independent biological replicates were used and are represented with an individual color each (red or blue). For each biological replicate, three technical repetitions of the germination percentage of 50–65 seeds are shown by a colored dot (red or blue). The average germination percentage for all the technical repetitions is represented by a horizontal black bar. (C) Protein gel blot analysis of phyA and PIF1 protein levels in WT, *phyA* and *phyBCDE* (*BCDE*) seeds exposed to a FR/12h/FR, FR/48h/FR or FR/96h/FR assay just before the second FR light pulse and thereafter as indicated. (D) Protein gel blot analysis of PIF1 protein levels in WT seeds exposed to a FR/R, FR/48h/FR or FR/96h/FR assay at the indicated time points after the R or second FR light pulse. For (A, C and D) UGPase protein levels were used as a loading control. All experiments were repeated with biological replicates, which provided similar results.

In a FR assay, which blocks WT seed germination, PIF1 levels rapidly increased between 1h and 6h (Fig 5A and S9 Fig), remained high between 6h and 24h and slowly decreased thereafter, consistent with previous reports [42]. In contrast, phyA levels slowly increased between 1h and 12h and remained roughly similar between 24h and 48h, consistent with previous reports [17], and further slowly increased between 48h and 96h (Fig 5A).

The phyA-mediated germination percentage of WT seeds exposed to a FR/12h/FR, and FR/48h/FR was 0% and 2%, respectively, whereas in seeds exposed to a FR/96h/FR assay it jumped to 37% despite the modest increase in phyA levels between 48h and 96h (Fig 5B). This is consistent with previous results showing that phyA levels and the percentage of phyA-mediated germination are not linearly correlated [17]. In contrast, and as expected, close to 100% phyB-mediated germination was observed in WT seeds exposed to a FR/R assay (Fig 5B).

We monitored endogenous PIF1 accumulation in seeds exposed to the different above FR/Nh/FR assays (N = 12, 48, 96), conducive of phyA-mediated germination. Irrespective of the time of its application, the second FR pulse did not affect markedly phyA protein levels (Fig 5C). Unexpectedly, however, the second FR pulse triggered, within one hour, rapid PIF1 downregulation in all assays (Fig 5C). As expected, endogenous PIF1 downregulation was not observed in *phyA* mutant seeds but observed in *phyBCDE* mutant seeds, showing that PIF1 downregulation is driven by phyA [43] (Fig 5C).

Thus, and surprisingly, FR and R light are similarly able to downregulate PIF1 levels even though they do not stimulate germination with the same efficiency. We therefore wondered whether the duration of PIF1 extinction time could be different after R and later FR light irradiation. Indeed, the duration of PIF1 extinction time was about 12h longer after a R pulse than after a second FR pulse in both a FR48hFR assay or a FR96hFR assay (Fig 5D).

Altogether these observations show that phyA levels in seeds are not limiting to promote PIF1 downregulation in response to a second FR light pulse applied at different times upon imbibition. They rather suggest that phyA-mediated germination is inefficient, at least in part, due to the short duration of PIF1 extinction following FR irradiation.

Interestingly, publicly available data indicate that expression of four polyamine biosynthesis genes is higher in *pif1* mutant seeds relative to WT seeds exposed to a FR assay [44] (S10A–S10D Fig). In contrast, expression of *PUT2* is low in *pif1* seeds relative to WT seeds exposed to a FR assay [44] (S10E Fig). Furthermore, expression of these genes was similar in WT and *pif1* seeds exposed to a FR/R assay (S10A–S10E Fig). This suggested that PIF1 represses PAs accumulation in seeds exposed to a FR assay.

**Fig 6:**
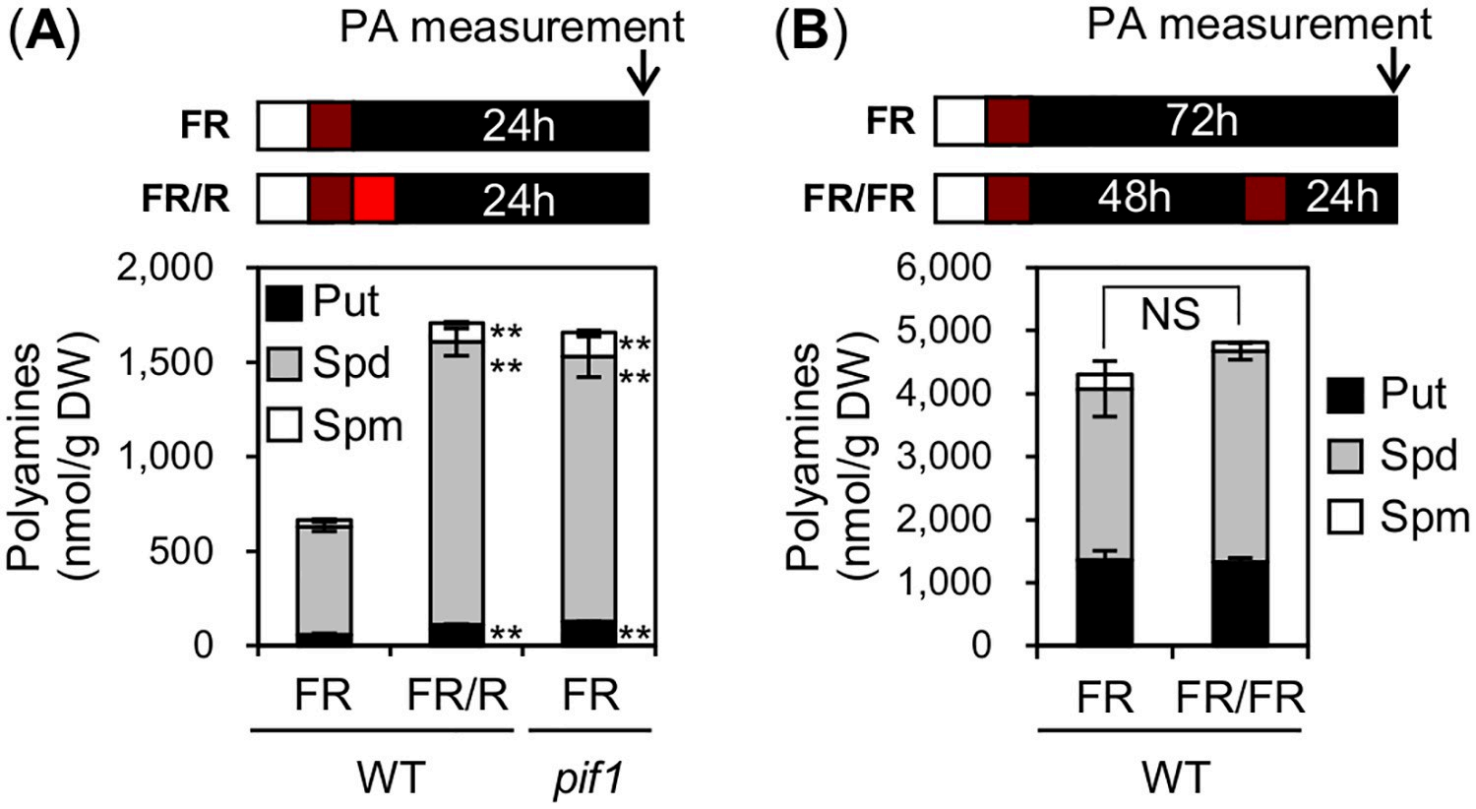
PIF1 represses PA accumulation in seeds. (A) Free PA (Put, Spd and Spm) levels in WT and *pif1* seeds exposed to a FR or FR/R assay. PAs were measured 24 h after the FR or R pulse. (B) Free PA (Put, Spd and Spm) levels in WT seeds exposed to a FR or FR/48h/FR assay. PAs were measured 72 h after the first FR pulse or 24 h after the second FR pulse. For (A and B) three technical repetitions were used for SD (n = 3). Statistical treatment as in Fig 1B. NS: Not Significant. Experiments were repeated with biological replicates, which provided similar results (S11 Fig).

To evaluate this possibility, we measured PAs levels in WT and *pif1* seeds exposed to a FR assay. Consistent with this hypothesis, PAs levels were higher in *pif1* mutant seeds relative to WT seeds exposed to FR assay (Fig 6A). These data are consistent with the view that PIF1 represses PAs accumulation in seeds after an early FR light pulse.

Next, we investigated whether PAs levels increase under conditions conducive of phyA-mediated germination. Unlike WT seeds exposed to a R pulse, PAs levels did not change after a second FR pulse relative to seeds exposed to a single FR pulse (Fig 6B and S11 Fig).

Altogether, these results suggest that the short duration of PIF1 extinction upon a second FR light pulse irradiation does not permit the elevation of PAs levels in seeds. As a result, low PAs levels would contribute to the low efficiency of phyA-mediated germination (see model below).

### *put2* and artificially aged seeds have enhanced phyA-mediated germination without changes in PIF1 levels

**Fig 7:**
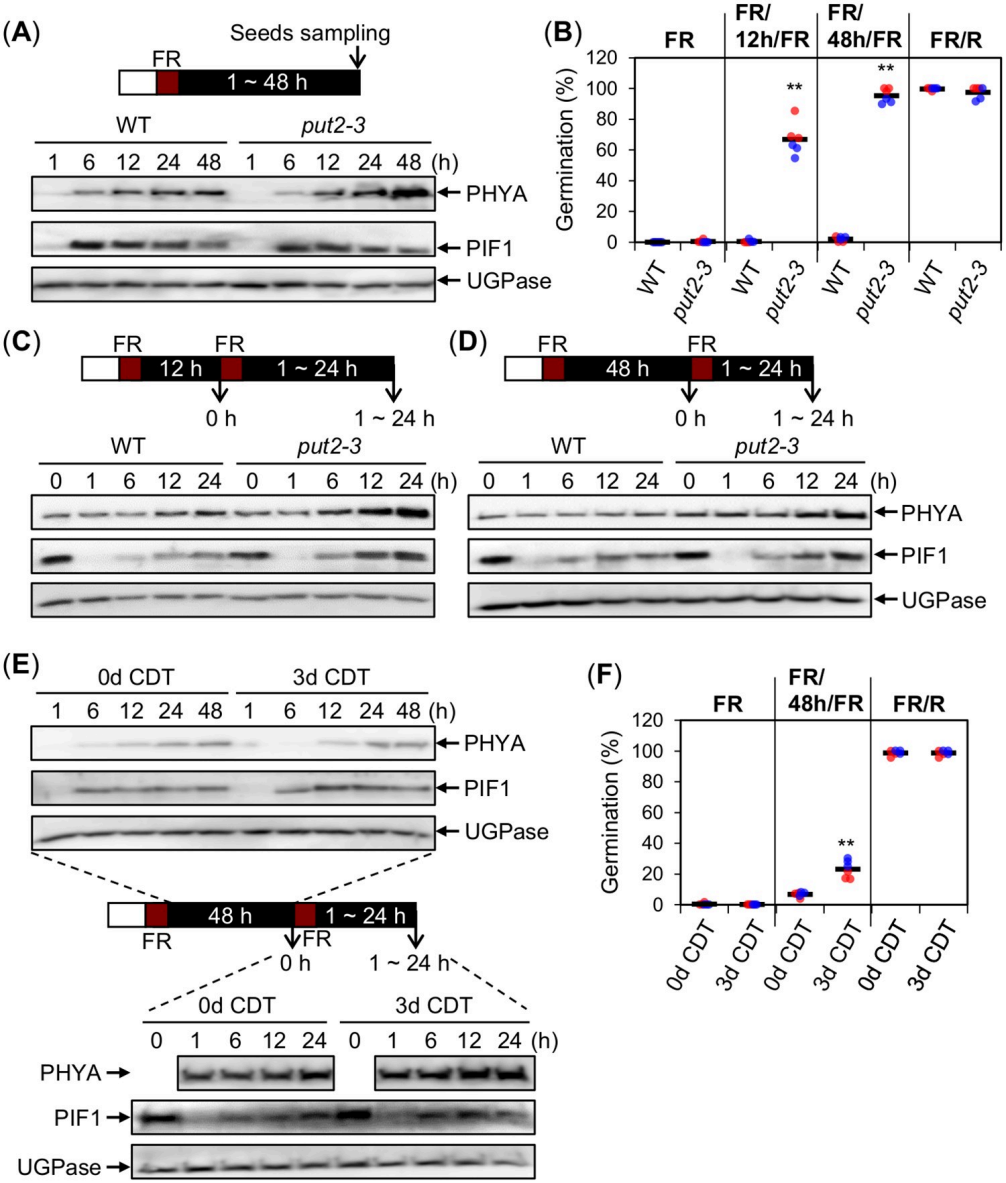
*put2* and artificially aged seeds have enhanced phyA-mediated germination without changes in PIF1 levels. (A) Protein gel blot analysis of phyA and PIF1 protein levels in WT and *put2-3* seeds exposed to a FR assay at the indicated time points after the FR light pulse. (B) Germination percentage of WT and *put2-3* seeds exposed to a FR, FR/12h/FR, FR/48h/FR or FR/R assay. (C and D) Protein gel blot analysis of phyA and PIF1 protein levels in WT and *put2-3* seeds exposed to a FR/12h/FR (C) or FR/48h/FR (D) assay just before the second FR light pulse and thereafter as indicated. Experiments were repeated with biological replicates, which provided similar results (S12B and S13B and S13C Figs). (E) Protein gel blot analysis of phyA and PIF1 protein levels in CDT-exposed WT seeds (0d and 3d) exposed to a FR/48h/FR assay before the second FR light pulse and thereafter as indicated. Experiments were repeated with biological replicates, which provided similar results (S14C and S14D Fig). (F) Germination percentage of CDT-exposed WT seeds (0d and 3d) exposed to a FR, FR/48h/FR or FR/R assay. For (A and C-E) UGPase protein levels were used as a loading control. For (B) and (F) two independent biological replicates were used and are represented with an individual color each (red or blue). For each biological replicate, three technical repetitions of the germination percentage of 50–65 seeds are shown by a colored dot (red or blue). The average germination percentage for all the technical repetitions is represented by a horizontal black bar. Statistical treatment as in Fig 1B.

We next investigated whether increased phyA-mediated germination in *put2* mutants or in WT seeds that had undergone CDT was linked to changes in phyA and PIF1 levels in seeds. In a FR assay, PIF1 levels in *put2-3* mutant seeds were similar to those in WT seeds (Fig 7A). *put2-3* mutant seeds accumulated normal phyA levels up to 12h after imbibition (Fig 7A). At 48h phyA levels were higher in *put2-3* seeds relative to WT seeds; we did not further investigate this matter.

Strikingly, a FR/12h/FR assay markedly stimulated germination of *put2-3* seeds relative to WT seeds (Fig 7B), even though they accumulated similar phyA levels (Fig 7C; 0 h and S12A and S12B Fig). Higher germination percentage of *put2-3* seeds was not associated with obvious differences in PIF1 levels, as assessed by data quantification in experiments with biological replicates, including in the duration of PIF1 extinction time after the second FR pulse (Fig 7C and S12A and S12B Fig). Furthermore, in a FR/48h/FR assay the duration of PIF1 extinction time after the second FR pulse was similar between WT and *put2-3* seeds even though the percentage of *put2-3* seed germination was 100% whereas that of WT seeds was only 2% (Fig 7B and 7D and S13A–S13C Fig).

After an early FR pulse, WT seeds that had undergone CDT had no obvious changes in phyA or PIF1 levels relative to untreated WT seeds up to 48h after FR light irradiation, as assessed by data quantification in experiments with biological replicates (Fig 7E and S14A Fig). After a second FR pulse (FR/48h/FR assay), the percentage of germination of WT seeds exposed to CDT was enhanced compared to unexposed WT seeds without obvious changes in phyA or PIF1 levels, as assessed by data quantification in experiments with biological replicates (Fig 7E and 7F and S14B–S14D Fig).

**Fig 8:**
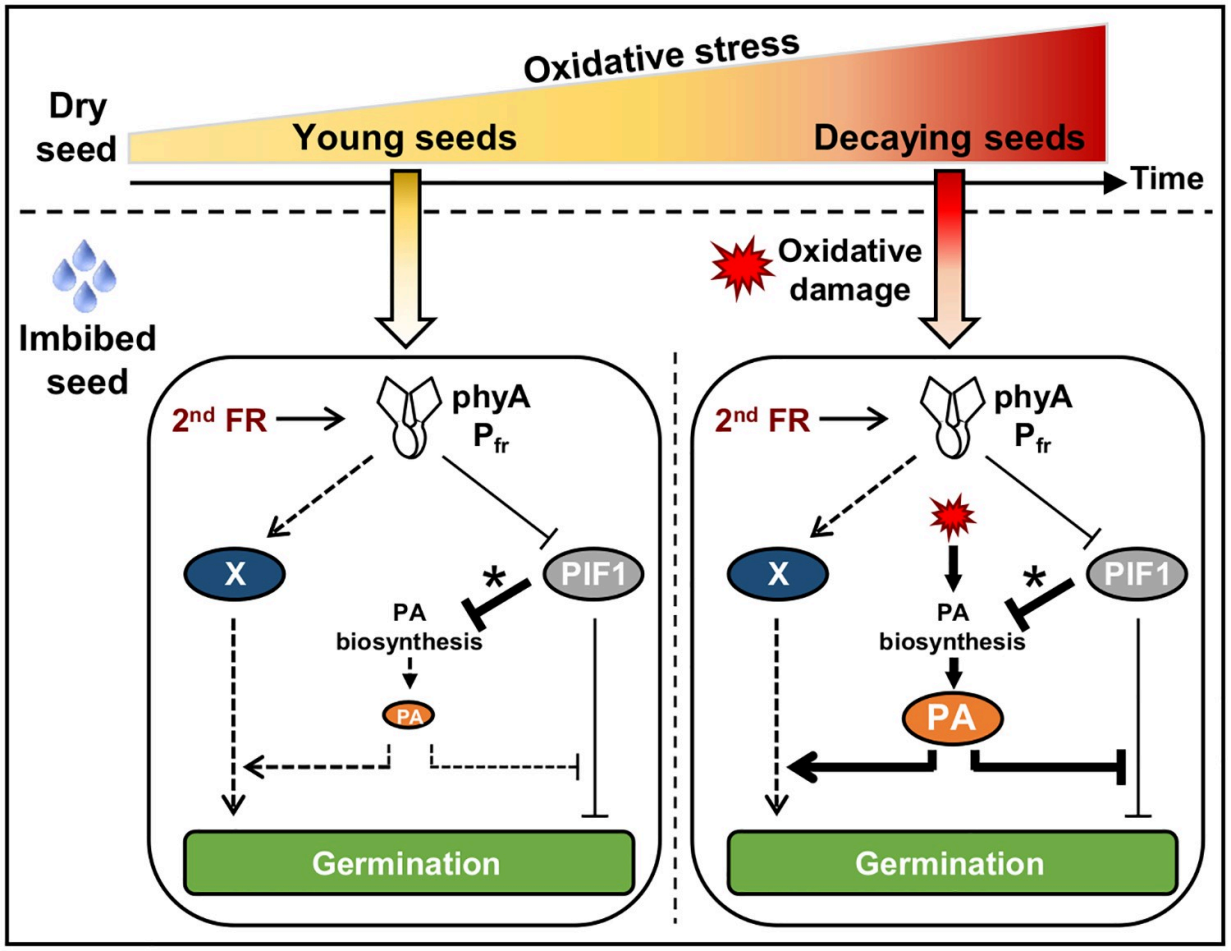
Model for phyA-mediated germination in young and decaying seeds. (Left panel) In younger seeds a second FR pulse late upon seed imbibition (2^nd^ FR) promotes PIF1 downregulation after activating phyA. However, PIF1 extinction time is short (asterisk), which maintains low PA levels and renders phyA-mediated germination less efficient. (Right panel) In decaying seeds, PIF1 extinction time after a second FR pulse remains short (asterisk). However, accumulation of oxidative damage (red symbol) leads to high PA levels thus bypassing PIF1 repression of PA accumulation. In turn, high PA levels enhance phyA-mediated germination. PAs could inhibit PIF1-dependent repression of germination or, alternatively, could promote phyA-mediated germination independently of PIF1 (putative “X” pathway). As seeds age, dry seeds decay due to accumulation of oxidative events, which compromises their capacity to form a viable seedling upon imbibition. In decaying seeds, oxidative stress promotes high PAs levels that could protect cells through their antioxidant activity while in parallel enhancing phyA-mediated seed germination as a last chance to survive even under unfavorable canopy light.

These observations show that enhanced phyA-mediated germination in *put2* seeds or WT seeds exposed to CDT can take place without marked changes in PIF1 levels. They therefore suggest that oxidative stress promotes PA accumulation, which in turn promotes phyA-mediated germination downstream of PIF1 or else independently of PIF1 (Fig 8).

## Discussion

Here we sought to better understand how seed germination is promoted by canopy light through phyA. We focused on understanding why this process is poorly efficient and its physiological relevance.

Downstream phyA signaling components in seeds are poorly characterized. Their identification and study is rendered difficult by the fact that observing phyA-mediated germination requires first blocking germination through phyB inactivation. In the case of *pif1* mutants, which fully germinate after an early FR pulse, germination mediated by phyA cannot be observed. Nevertheless, it is generally assumed that PIF1 represses germination downstream of phyA [23,43].

Here we found that *put2* mutants are specifically enhanced in phyA-mediated germination (Figs 1 and 2), which was not previously reported. *PUT2* encodes a PAs transporter and we showed that among other mutants deficient in homologous transporters only *put2* mutants accumulate high PAs levels in seeds (Fig 3). Similarly, mutants deficient in PAs catabolism or WT seeds exposed to CDT increased PAs levels in seeds and enhanced phyA-mediated germination (Figs 3 and 4). Thus, our study provides correlative evidence suggesting that increased PAs levels in seeds positively regulate phyA-mediated germination.

High PAs accumulation in *put2* seeds could result from inappropriate distribution of PAs in cells. PUT2 is localized in the Golgi apparatus and the chloroplast [24,45]. A *put2* mutant cell sensing low PAs accumulation in the Golgi or chloroplast might respond with increased PAs synthesis as a compensatory mechanism. *put2* had increased Spd and Spm levels whereas WT seeds exposed to CDT and *pao2pao4* mutants had mainly higher Spd levels. Furthermore, PUT2 preferentially transports Spd in yeast cells [29]. This could suggest that Spd is important to enhance phyA-mediated germination. However, exogenous application of Put, Spd and Spm similarly promoted phyA-mediated germination (Fig 3). Thus, our study does not pinpoint a particular individual PA specifically promoting phyA-mediated germination. Understanding the role of an individual PA in phyA signaling using genetic approaches is difficult since, to our best knowledge, there are no reported PA biosynthesis mutants accumulating a single PA.

It remains to be understood how PAs promote phyA signaling in seeds. L-arginine is a precursor of Put and S-adenosylmethionine is a precursor of Spd and Spm. However, they are also precursors of nitric oxide (NO) and ethylene, respectively. Indeed, enhanced PAs accumulation was linked to an increase in NO levels and associated with both an increase and decrease in ethylene signaling [46–48]. NO and ethylene repress ABA responses in seeds and therefore PAs could enhance germination by repressing ABA signaling through NO or ethylene [49]. Against this possibility, we found that *put2* mutants have normal ABA responses in seeds, consistent with a previous report showing that *par1* mutants have normal ABA responses [24] (S15 Fig). PAs are involved in a wide range of fundamental cellular processes including DNA replication, transcription, translation and post-translational modification [7]. PAs were also speculated to participate in abiotic stress signaling in plants [50]. In all cases, the mechanism by which PAs act is poorly understood as they are difficult to study due to their ubiquitous presence in cells and their essential function for survival [6].

Our data suggest that endogenous PAs accumulation is repressed by PIF1 in WT seeds exposed to an early FR pulse. This indicates that PAs biosynthesis is regulated downstream of PIF1. The biological significance of the regulation of PAs biosynthesis gene expression and PAs levels in seeds by light remains to be understood. On the other hand, PAs levels were higher in *put2* mutants or in WT seeds exposed to CDT and the resulting increase in phyA-mediated germination took place without changes in PIF1 levels. Therefore, this suggests that PAs levels can be regulated independently of PIF1 to regulate phyA-mediated germination. PAs could promote phyA-mediated germination downstream of PIF1. However, transcriptomic studies have suggested that phyA activation by FR also triggers gene expression changes independently of PIF1 [18]. Thus, PAs could promote phyA-mediated germination in a PIF1-independent manner (Fig 8; see putative “X” pathway in the model).

PAs levels in seeds increased in response to R light but not in response to a second FR light pulse (Fig 6). We propose that this is due to the short PIF1 extinction time following FR irradiation. This shorter time was not due to limiting phyA levels since it remained unchanged in WT seeds exposed to a FR/96h/FR assay or in *put2* seeds exposed to a FR/48h/FR assay, which had higher phyA levels (Figs 5 and 7). Therefore, PIF1 reaccumulation is differently regulated after R and FR light irradiation, respectively. The underlying mechanism accounting for this differential regulation remains to be identified. It is also unknown why *put2* mutants accumulate higher phyA levels at later time points upon seed imbibition.

It is generally accepted that PAs act as antioxidants in plants [51]. Seed oxidation is an unavoidable process compromising seed viability in the dry seed state. It is therefore expected that seeds have evolved adaptive mechanisms to sense oxidative damage and adapt their behavior such as their control of seed germination. In newly produced seeds, the first level of germination control is that of primary seed dormancy, a trait whereby germination is blocked even under favorable conditions. Dormancy prevents germination out of season. Seeds lose dormancy during a period of dry storage called dry after-ripening [2]. Seed oxidation is known to accelerate the release of seed dormancy during after-ripening [1]. Dormancy was shown to inhibit R- and phyB-mediated germination [52] and therefore it is expected that oxidation promotes phyB-mediated germination. However, the oxidation events that release dormancy are not sufficient to promote germination mediated by phyA: 18-month-old WT seeds, which have fully lost dormancy and fully germinated after a R pulse, germinated at less than 5% in a FR/48h/FR assay (Fig 2).

Our results therefore suggest that there are two levels of seed germination regulation through oxidative stress. Younger and still viable seeds, with moderate levels of oxidative damage, have an advantage to repress their germination under canopy light, which is unfavorable for photosynthesis. As seeds age, however they decay as a result of continuous accumulation of oxidative damage, which compromises their capacity to form a viable seedling. It would then become advantageous to germinate under a broader range of light wavelengths, including canopy light [19]. In this context, increased PAs levels upon seed imbibition could serve a dual function: protect the decaying seed from the oxidative damage that accumulated during dry after-ripening while promoting in parallel phyA-mediated germination under unfavorable light cues such as canopy light. This would represent a mechanism providing a last chance for plant survival (Fig 8).

## Materials and methods

### Plant materials

Arabidopsis T-DNA insertion lines, all in the Col-0 background, were obtained from the Nottingham Arabidopsis Stock Centre with the following accession numbers: *put2-3*; SALK_119707, *put1*; SAIL_270_G10, *put3*; SALK_206472, *put4*; SAIL_1275_C06, *put5*; SALK_122097, *pao1-2*; SAIL_882_A11, *pao2-4*; SALK_046281, *pao3-1*; GABI_209F07, *pao4-1*; SALK_133599, *pao5-2*; SALK_053110 and *phyA-211*; N6223. *phyA-211put2-3* double mutants were generated after crossing *phyA-211* and *put2-3* plants. *par1-1* [24], *pao1pao5* [37], *pao2pao4* [37] and *phyBCDE* [53] seeds were kindly provided by Jianru Zuo (Chinese Academy of Sciences, China), Tomonobu Kusano (Tohoku University, Japan) and Pablo D. Cerdán (Fundación Instituto Leloir, Argentina), respectively.

### Germination assays

All genotypes tested in each experiment were grown together under the same conditions and seeds were harvested the same day and allowed to after-ripen at room temperature for at least one month. For the germination assays, seeds were surface sterilized and 50–60 seeds of each genotype were sown on MS medium (Sigma) containing 0.8% (w/v) agar without seed stratification. For the germination assays in presence of polyamines, individual polyamines (Sigma) were added to the germination medium. In a FR assay, seeds were irradiated with a FR pulse (3.69 μmol m^-2^ s^-1^) for 5 min after 2 h seed imbibition under white light. In a FR/Nh/FR assay, seeds were irradiated with a first FR pulse (3.69 μmol m^-2^ s^-1^) for 5 min and further irradiated with a second FR pulse (3.69 μmol m^-2^ s^-1^ or as indicated in each experiment) for 5 min after N (e.g. 12, 48 and 96) hours of dark incubation. In a FR/R assay, seeds were irradiated with a red (R) pulse (14.92 μmol m^-2^ s^-1^) for 5 min followed by a FR pulse. In all assays, light irradiated plates were kept in the dark for the indicated times. Thereafter a seed that had undergone endosperm rupture, i.e. radicle protrusion, was scored as a germination event. All the germination assays were performed with three technical replicates and the results were confirmed with at least two or three independent biological seed samples. Data value distribution among biological samples is shown by scatterplots as described in Weissgerber *et al*. (2015) [54].

### Generation of transgenic plants and genetic screen

Approximately 3kb of *GA3ox1* promoter region [20] was amplified with primers (5’-CGCGGATCCCACCAGAGTGTGTGCTACATGC-3’ and 5’-CCGCTCGAGAACACAGCAGGCAGCTTGCTC-3’). BamHI and XhoI restriction sites were used for cloning into binary vector pGPTVII [55]. WT (Col-0) plants were transformed with a firefly luciferase (*LUC*) reporter gene under the control of *GA3ox1* promoter sequences (WT/*pGA3ox1*::*LUC*). With the aim of identifying mutants displaying enhanced phyA-mediated seed germination responses, a population of 20,000 WT/*pGA3ox1*::*LUC* seeds (M0) was chemically mutagenized using 0.3% ethyl methanesulfonate (EMS) as previously described [56]. In M2 populations, mutants able to germinate in a FR/12h/FR assay or displaying high LUC bioluminescence were selected for further analysis (Fig 1 and S1 Fig). LUC bioluminescence was performed as previously described [55]. Briefly, plants exposed to FR/12h/FR assay were sprayed with a luciferin (Biosynth) solution (315 μg/ml), under green safety light, 24h after the second FR pulse and examined using an Aequoria dark box with a mounted ORCAII CCD camera (Hamamatsu). This led to identify three recessive and independent mutants (*ffg1*—*ffg3*) having enhanced bioluminescence and germination in a FR/12h/FR assay relative to the parental non-mutagenized WT/*pGA3ox1*::*LUC* line. The same mutagenized seed population was used to identify *put2-2* as previously described [24,25]. Briefly, the same mutagenized seed population was sown on a germination medium with 0 and 10 μM of PQ and cultured under WLc for 6 days to reveal PQ-insensitive mutants, which led to the identification of the *put2-2* allele.

### Molecular mapping and whole genome sequencing

For map-based cloning and whole genome sequencing, the *ffg1* mutant was outcrossed to Ler. The *ffg1* locus was mapped as previously described [57]. Briefly, a combination of cleaved amplified polymorphic sequences (CAPS) markers and simple sequence length polymorphisms (SSLPs) markers was used for fine mapping. The *ffg1* locus was mapped to a 200 kbp interval on chromosome 1 (11.3 ~ 11.5 Mbp). Identification of mutations in this interval was done after sequencing the *ffg1* mutant genome. Genomic library preparation was performed using TrueSeq^®^ DNA Library Prep Kit (Illumina) according to manufacturer’s instructions. Sequencing was performed using HiSeq2000 (Illumina). This led to the identification the *put2-1* allele in the 200 kbp interval.

### Measurement of free polyamines (PAs) levels

To avoid detecting differences in PAs levels arising from plants at different development stages, we measured endogenous PAs levels in dry or non-germinated seeds that are harvested under green safety light. Standards of polyamines as well as the other chemicals and reagents were purchased from Sigma Aldrich chemical company (St. Louis, MO, USA). Free PAs were isolated and derivatized by slightly modified method of previous report [58]. A 250 mL of 5% trichloroacetic acid (TCA) was added to a 5 mg of lyophilized seeds and homogenized using ZrO_2_ beads (3 mm) in mixer-mill for 5 min at 27 Hz. Sample was then sonicated for 10 min at 25 °C, and after centrifugation at 12,400 × g for 5 min, supernatant was quantitatively transferred into another vial.

A 500 μL of 2M NaOH was added following with 2.5 μL of benzoyl chloride (in methanol 50:50, v:v), and after vortexing for 5 sec reaction mixtures are left for 40 min at 25 °C. A 500 μL of saturated NaCl was added and benzoylated polyamines were extracted with 2 × 500 μL of diethyl ether. Ether was evaporated and dry samples were stored at -80 °C until analysis.

All samples were dissolved in 50 μL of mobile phase (45% methanol in 15 mM formic acid, pH 3.0), sonicated for 15 min, and centrifuged for 5 min at 12,400 × g prior to the analysis. Diaminohexane (DAH) was used as internal standard.

Ultra-high performance liquid chromatography-tandem mass spectrometry (UHPLC-MS/MS) was performed on UltiMate^™^ 3000 liquid chromatographic system consisting of binary pumps, an autosampler and a column thermostat coupled to a TSQ Quantum Access Max triple quadrupole mass spectrometer (Thermo Fisher Scientific, Waltham, MA, USA).

Chromatographic separation was performed on an Acquity UPLC BEH C18 (50 × 2.1 mm; 1.7 μm particle size) column (Waters, Milford, MA, USA) with appropriate pre-column kept at 40 °C. The mobile phase consisted of a mixture of aqueous solutions of 15 mM formic acid adjusted pH 3.0 with ammonium hydroxide (Solvent A) and methanol (Solvent B). The analytes were separated using a binary gradient starting at 45% of B for 2.7 min, then increase to 57% for 0.3 min, isocratic at 57% for next 2.5 min, increase to 100% B for next 0.1 min, isocratic at 100% B for next 1 min, and decrease to 45% B for next 0.1 min. Finally, the equilibration to the initial conditions took 2.3 min. The flow rate was 0.4 mL/min and the injection volume 5 μL. Benzoylated PAs were detected in positive ionization mode electrospray ionization (ESI+). The selected reaction monitoring (SRM) transitions for benzoylated putrescine (Put) were 297 > 105, and 297 > 176 at 20 eV collision energy (CE), for spermidine (Spd) 458 > 175, and 458 > 233 at 25 eV CE, and for spermine (Spm) 619 > 162, 619 > 337, and 619 > 497 at 30 eV CE. The spray voltage was set to 3 kV, the vaporizer temperature to 350 °C, and the ion transfer tube temperature to 320 °C, respectively.

### Controlled deterioration treatment (CDT)

CDT was performed as described in a previous report [4]. Briefly, Col-0 dry seeds were stored in a closed container during the time period indicated in each experiment. The container was maintained at 37 °C and its interior had around 82% of relative humidity imposed by the presence of a saturated salt (KCl). Then, seeds were dried back at 30% relative humidity (room temperature) and stored at -80 °C until they were used for superoxide O_2_^-^ levels measurements, germination assays or protein gel blots analysis.

### Determination of superoxide O_2_^-^ levels

The levels of superoxide O_2_^-^ were determined based on its ability to reduce nitro blue tetrazolium (NBT) to blue formazan as previously described [59,60]. Dry seeds (0.035g) were grinded in 1.3 mL of incubation solution (10 mM K-phosphate buffer pH 7.8, 10 mM NaN_3_, 0.05% NBT). After 30 min of incubation at room temperature, grinded tissue was collected at the bottom of the tube by centrifugation (8000 x g, 5 min, room temperature). Supernatant was diluted 10 x in incubation buffer, heated 85 °C for 15 min and then cooled on ice. Absorbance at 580 nm was measured by spectrophotometer to quantify NBT levels.

### Antibody production and protein gel blot analysis

PIF1 recombinant proteins were prepared using PIF1-his DNA (pET21a) provided by Enamul Huq (University of Texas at Austin, USA), and induced and purified using a commercial kit according to manufacturer’s instructions (Amersham). Polyclonal anti-PIF1 was obtained from rabbits immunized with PIF1 recombinant protein. PIF1 antibodies were further affinity-purified using PIF1 recombinant protein immobilized on nitrocellulose filters as described [61]. FR or R light irradiated WT and mutant seeds were harvested under green safety light at the time indicated in each experiment. 20 seeds were homogenized with homogenization buffer (0.0625 M Tris-HCl at pH 6.8, 1% [w/v] SDS, 10% [v/v] glycerol, 0.01% [v/v] 2-mercaptoethanol), and total proteins were separated by SDS-PAGE gel and transferred to a PVDF membrane (Amersham). PIF1 and UGPase proteins were detected using 1:500 dilution of anti-PIF1 or 1:10,000 dilution of anti-UGPase (Agrisera), and anti-rabbit IgG HRP-linked whole antibody (GE healthcare) in a 1:10,000 dilution was used as a secondary antibody. PHYA proteins were detected using 1:5,000 dilution of anti-phyA (kindly provided by Akira Nagatani; Kyoto University, Japan) and the anti-mouse IgG HRP-linked whole antibody (GE healthcare) in a 1:10,000 dilution was used as a secondary antibody [17]. Quantification of band intensity was performed using imageJ.

## Supporting information

S1 Fig *pif1* and *aba1* mutant identified in a genetic screen. (A) Pictures show luminescence of WT(Col-0)/*pGA3ox1*::*LUC* (Parent.) and *ffg* mutant seeds exposed to a FR/12h/FR assay 24h after second FR light pulse. (B-D) Histograms show germination percentages of Parent., *phyA*, *ffg4*(*pif1*) and *ffg5*(*aba1*) seeds exposed to a FR (B), FR/12h/FR (C) or FR/R (D) assay. One biological seed batch sample was used to measure the average seed germination percentage using three technical repetitions. For each repetition, the germination percentage of 50–65 seeds is shown. Statistical treatment as in Fig 1B. (E and F) Diagrams show the genomic structure of *PIF1* (E) and *ABA1* (F) genes with location of mutations identified in the genetic screen. Black boxes: exons, black lines: introns, white boxes: UTRs and arrows: transcription start sites. Bar: 100 bp.
(TIF)

S2 Fig Paraquat (PQ) sensitivity of WT and *put2* mutant and protein gel blot analysis of *phyA-211put2-3* double mutant seeds. (A) Pictures show wild type (Parent. and Col-0) and different *put2* mutant allele plants cultured in absence or presence of paraquat (PQ), as indicated, for 7 days. (B) Protein gel blot analysis of phyA protein levels in WT, *phyA-211*, *put2-3* and *phyA-211put2-3* seeds harvested 48h after a FR light pulse (FR assay). UGPase protein levels were used as a loading control.
(TIF)

S3 Fig Alignment of PUT1–PUT5 amino acid sequences by ClustalW. Alignment of PUT1—PUT5 amino acid sequences by ClustalW. “*” fully conserved residue, “:” fully conserved strong groups (STA, NEQK, NHQK, NDEQ, QHRK, MILV, MILF, HY and FYW), “.” fully conserved weaker groups (CSA, ATV, SAG, STNK, STPA, SGND, SNDEQK, NDEQHK, NEQHRK, FVLIM and HFY).
(TIF)

S4 Fig Expression of *PUT1*–*PUT5* in developing, mature dry and imbibed seeds. Expression of *PUT1*—*PUT5* during seed development, mature dry seed and seed imbibition. Data were extracted from BAR (http://www.bar.utoronto.ca/).
(TIF)

S5 Fig T-DNA insertions of *put1*, *put3*, *put4* and *put5*. Diagram shows the genomic structure of *PUT1*, *PUT3*, *PUT4* and *PUT5*. The location of the T-DNA insertions used in this study are shown. Black boxes: exons, black lines: introns, white boxes: UTRs and arrows: transcription start sites. Bar: 100 bp.
(TIF)

S6 Fig Free PA levels in WT and *put* seeds. (A) Free PA (Put, Spd and Spm) levels in four different Col-0 (WT) seed batches exposed to a FR assay at the indicated time point. (B) Free PA (Put, Spd and Spm) levels in two different seed batches of WT and *put2-3* mutant exposed to a FR assay at the indicated time point. (C) Free PA (Put, Spd and Spm) levels in WT and *put1*—*put5* dry seeds. (D) Free PA (Put, Spd and Spm) levels in Parent., WT and different *put2* mutant seeds exposed to a FR assay at the indicated time point. For (A-D) three technical repetitions were used for SD (n = 3). Statistical treatment as in Fig 1B.
(TIF)

S7 Fig Superoxide and free PA levels of CDT-exposed WT seeds. (A) Determination of superoxide O_2_^-^ levels in CDT-exposed WT dry seeds. Four different seed batches were used for the assay and depicted in the graph. (B) Free PA (Put, Spd and Spm) levels in two different seed batches of CDT-exposed WT seeds. PAs were measured 24 h after the second FR pulse. Three technical repetitions were used for SD (n = 3). Statistical treatment as in Fig 1B.
(TIF)

S8 Fig Germination of CDT-exposed WT seeds. (A) Picture shows different days of CDT-exposed WT seeds grown under constant white light (WLc) for 72 h. (B and C) Germination percentage of two different seed batches of CDT-exposed WT seeds in a FR, FR/48h/FR or FR/R assay. For each repetition, the germination percentage of 50–65 seeds is shown by a blue (B) or green (C) dot. The average germination percentage for the three repetitions is represented by a horizontal black bar. Statistical treatment as in Fig 1B.
(TIF)

S9 Fig Specificities of phyA and PIF1 antibodies. Protein gel blot analysis of phyA protein levels in WT and *phyA* seeds (left), and PIF1 protein levels in WT and *pif1* seeds (right) exposed to a FR assay at the indicated time point after the FR pulse. UGPase protein levels were used as a loading control.
(TIF)

S10 Fig mRNA expression of PA biosynthesis genes and *PUT2*. (A-E) Publicly available microarray expression data of PA biosynthesis genes: *ARGININE DECARBOXYLASE 1*; *ADC1* (A), *AGMATINE IMINOHYDROLASE*; *AIH* (B), *SPERMIDINE SYNTHASE 1*; *SPDS1* (C), *S-ADENOSYLMETHIONINE DECARBOXYLASE 2*; *SAMDC2* (D), and *PUT2* (E) in WT and *pif1* seeds exposed to a FR or FR/R assay. Microarray data were provided by Dr. Giltsu Choi. Two technical repetitions were used for SD (n = 2). Statistical treatment as in Fig 1B.
(TIF)

S11 Fig Free PA levels in WT seeds exposed to a FR or FR/48h/FR assay. Free PA (Put, Spd and Spm) levels in two different seed batches of WT seeds exposed to a FR or FR/48h/FR assay. PAs were measured 72 h after the first FR pulse or 24 h after the second FR pulse. Three technical repetitions were used for SD (n = 3). Statistical treatment as in Fig 1B. NS: Not Significant.
(TIF)

S12 Fig Quantification and repetition of Fig 7C. (A) Quantification of Fig 7C. Accumulation of phyA and PIF1 protein levels is shown. (B) Biological replicates of Fig 7C and accumulation of phyA and PIF1 protein levels is shown.
(TIF)

S13 Fig Quantification and repetition of Fig 7D. (A) Quantification of Fig 7D. Accumulation of phyA and PIF1 protein levels is shown. (B and C) Biological replicates of Fig 7D and accumulation of phyA and PIF1 protein levels is shown.
(TIF)

S14 Fig Quantification and repetition of Fig 7E. (A and B) Quantification of Fig 7E upper (A) and bottom (B). Accumulation of phyA and PIF1 protein levels is shown. (C and D) Biological replicates of Fig 7E and accumulation of phyA and PIF1 protein levels is shown.
(TIF)

S15 Fig ABA sensitivity of WT and *put2* mutant seeds. Graph shows germination percentage of WT and *put2-3* mutant seeds in absence or presence of different concentrations of ABA. Seeds were grown under constant white light and germination percentage (radicle protrusion) was scored at the indicated time points.
(TIF)
