# Supplementary figures and images for: polyamine uptake transporter 2 (put2) and decaying seeds enhance phyA-mediated germination by overcoming PIF1 repression of germination
Source: PLoS Genet. 2019 Jul 24;15(7):e1008292. doi: 10.1371/journal.pgen.1008292 (PMC6682160; doi:10.1371/journal.pgen.1008292)

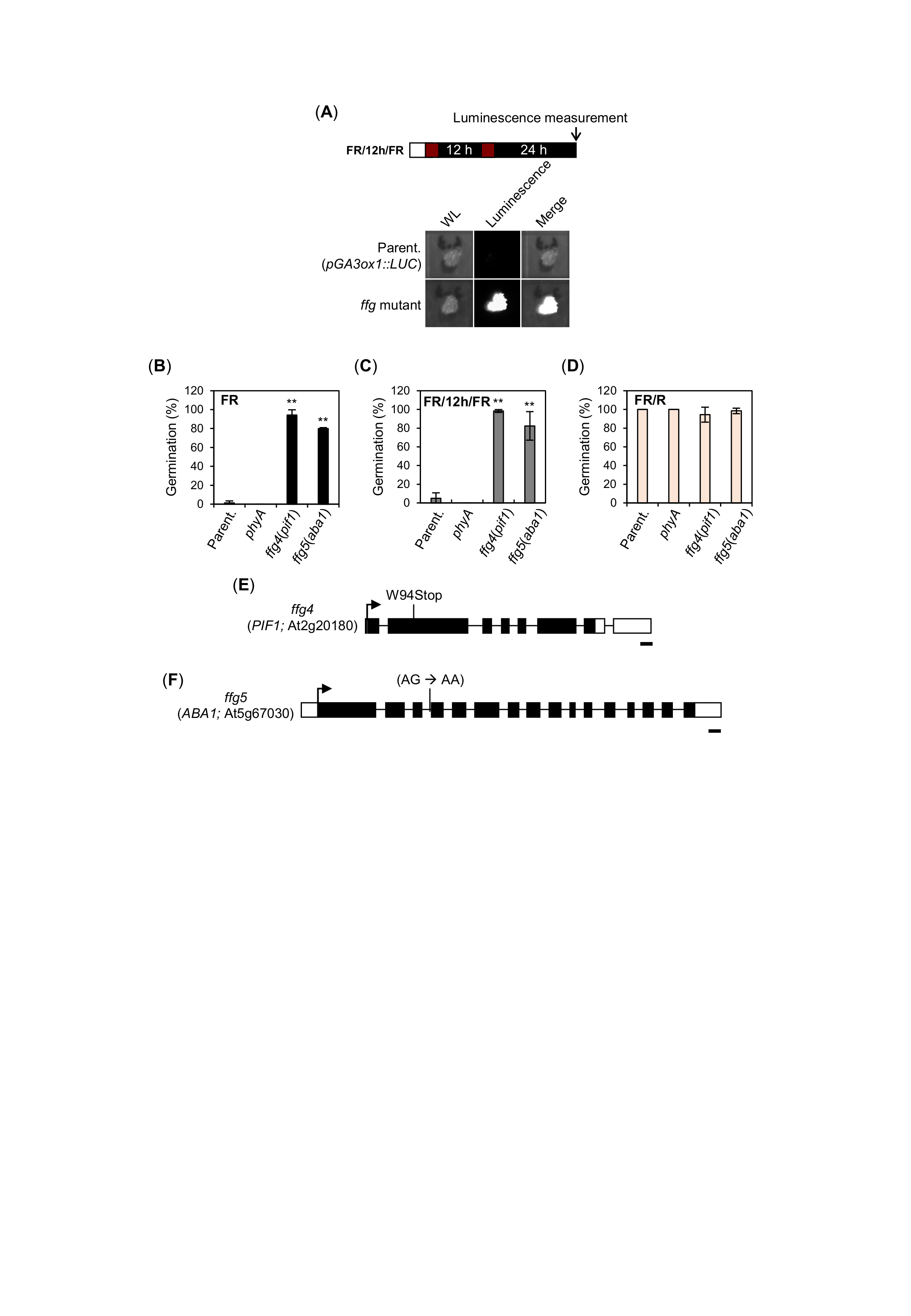

Supplement: S1 Fig — (A) Pictures show luminescence of WT(Col-0)/pGA3ox1::LUC (Parent.) and ffg mutant seeds exposed to a FR/12h/FR assay 24h after second FR light pulse. (B-D) Histograms show germination percentages of Parent., phyA, ffg4(pif1) and ffg5(aba1) seeds exposed to a FR (B), FR/12h/FR (C) or FR/R (D) assay. One biological seed batch sample was used to measure the average seed germination percentage using three technical repetitions. For each repetition, the germination percentage of 50–65 seeds is shown. Statistical treatment as in Fig 1B. (E and F) Diagrams show the genomic structure of PIF1 (E) and ABA1 (F) genes with location of mutations identified in the genetic screen. Black boxes: exons, black lines: introns, white boxes: UTRs and arrows: transcription start sites. Bar: 100 bp. (TIF) [file pgen.1008292.s001.tif]

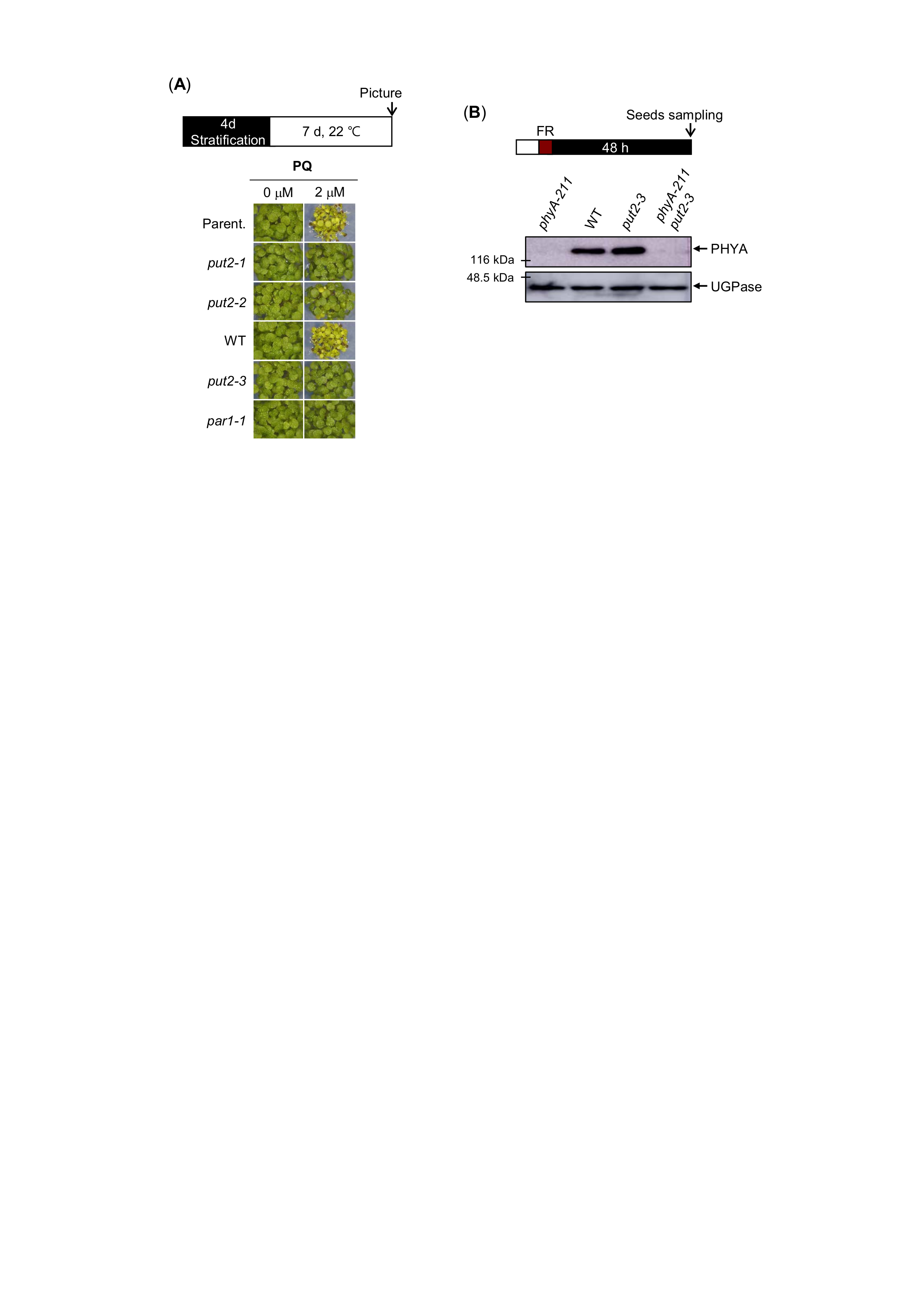

Supplement: S2 Fig — (A) Pictures show wild type (Parent. and Col-0) and different put2 mutant allele plants cultured in absence or presence of paraquat (PQ), as indicated, for 7 days. (B) Protein gel blot analysis of phyA protein levels in WT, phyA-211, put2-3 and phyA-211put2-3 seeds harvested 48h after a FR light pulse (FR assay). UGPase protein levels were used as a loading control. (TIF) [file pgen.1008292.s002.tif]

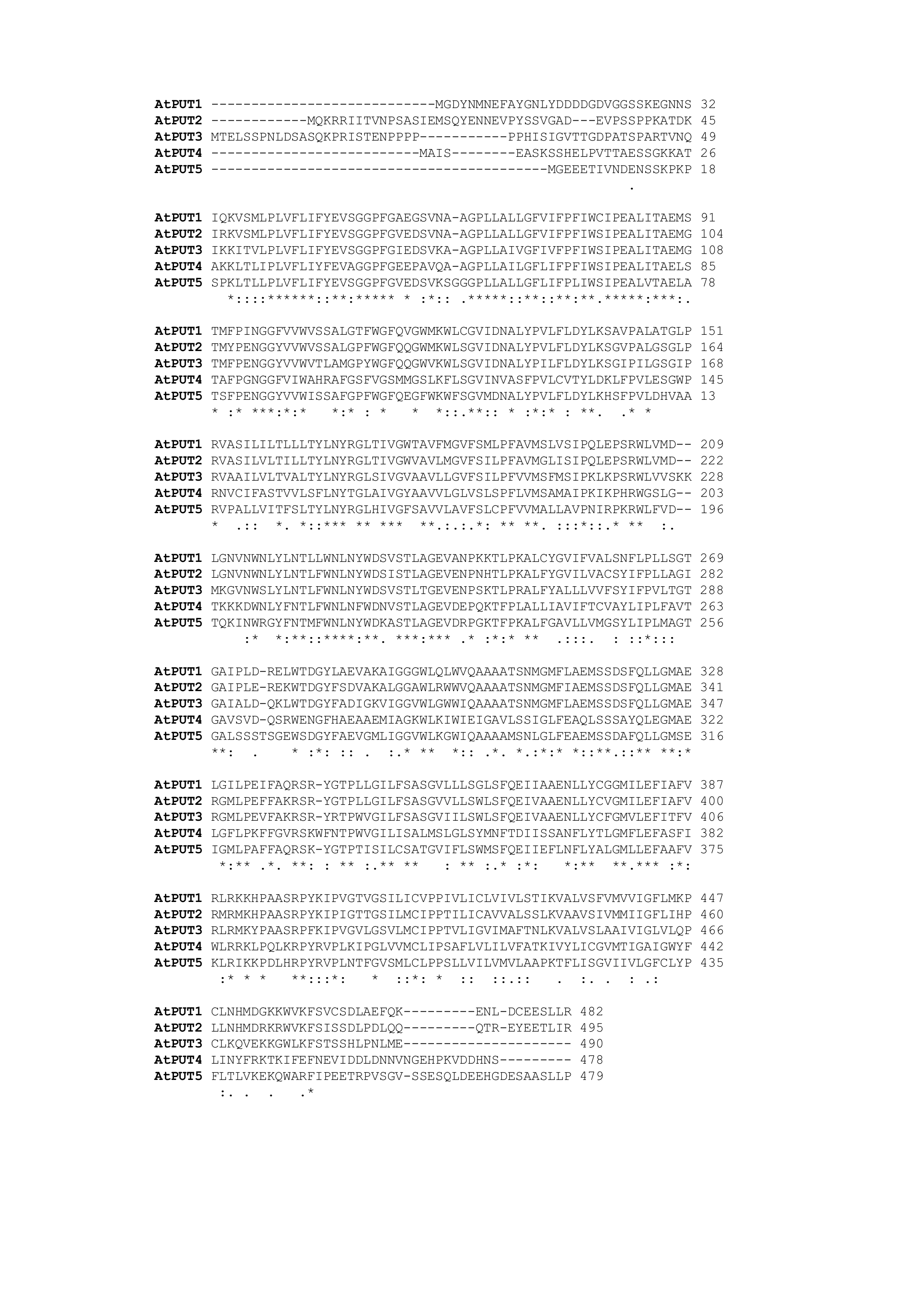

Supplement: S3 Fig — Alignment of PUT1—PUT5 amino acid sequences by ClustalW. “*” fully conserved residue, “:” fully conserved strong groups (STA, NEQK, NHQK, NDEQ, QHRK, MILV, MILF, HY and FYW), “.” fully conserved weaker groups (CSA, ATV, SAG, STNK, STPA, SGND, SNDEQK, NDEQHK, NEQHRK, FVLIM and HFY). (TIF) [file pgen.1008292.s003.tif]

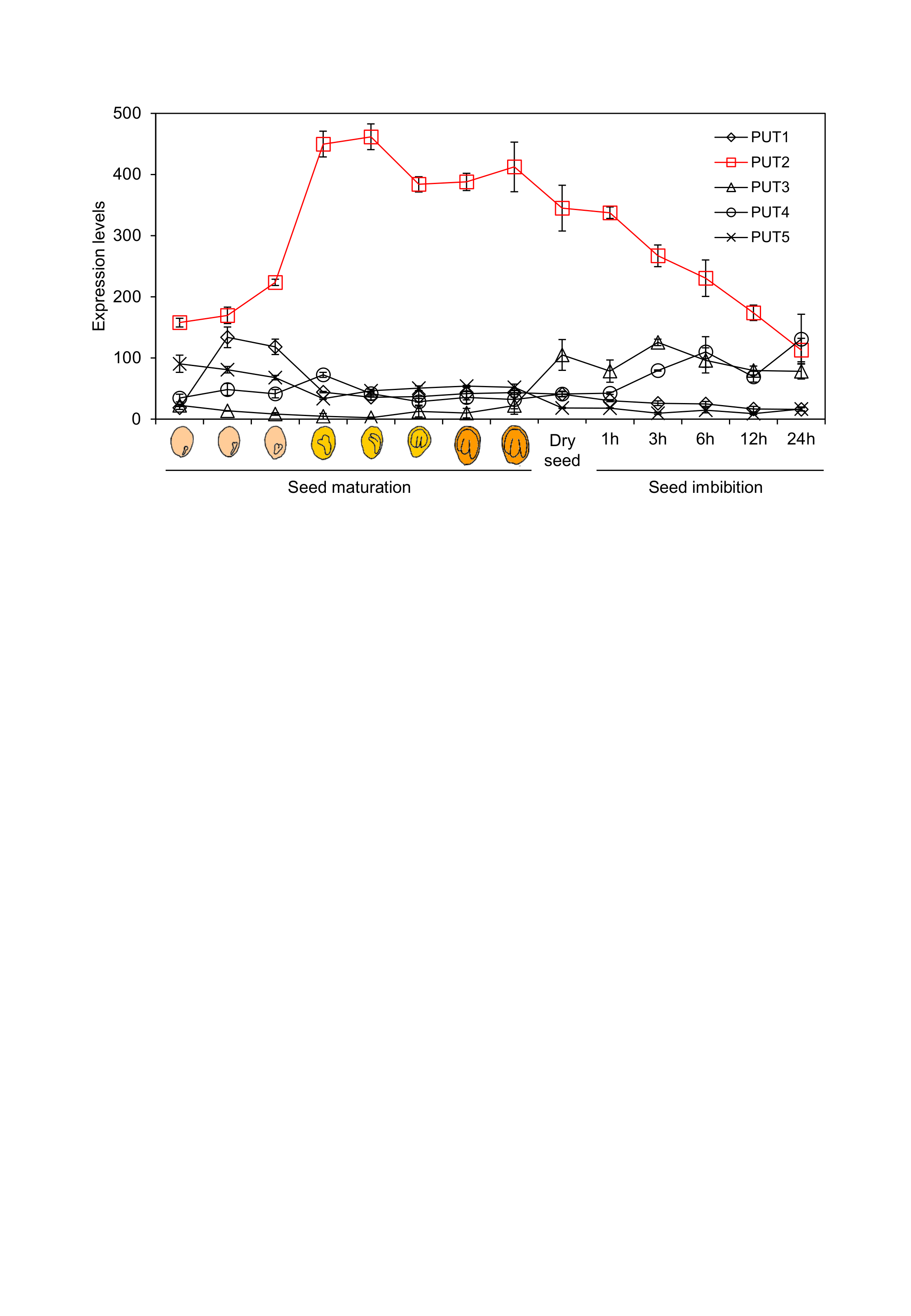

Supplement: S4 Fig — Expression of PUT1—PUT5 during seed development, mature dry seed and seed imbibition. Data were extracted from BAR (http://www.bar.utoronto.ca/). (TIF) [file pgen.1008292.s004.tif]

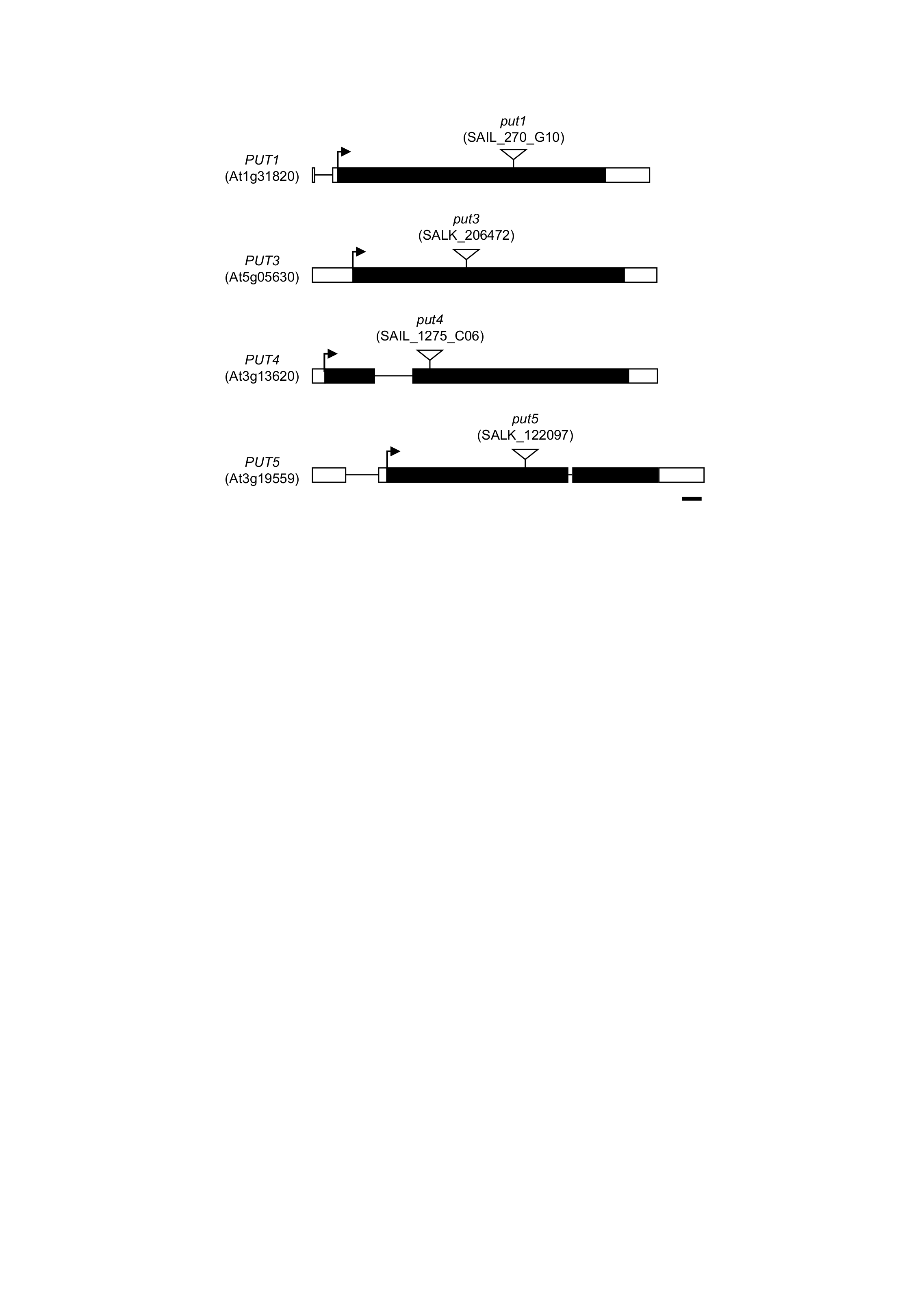

Supplement: S5 Fig — Diagram shows the genomic structure of PUT1, PUT3, PUT4 and PUT5. The location of the T-DNA insertions used in this study are shown. Black boxes: exons, black lines: introns, white boxes: UTRs and arrows: transcription start sites. Bar: 100 bp. (TIF) [file pgen.1008292.s005.tif]

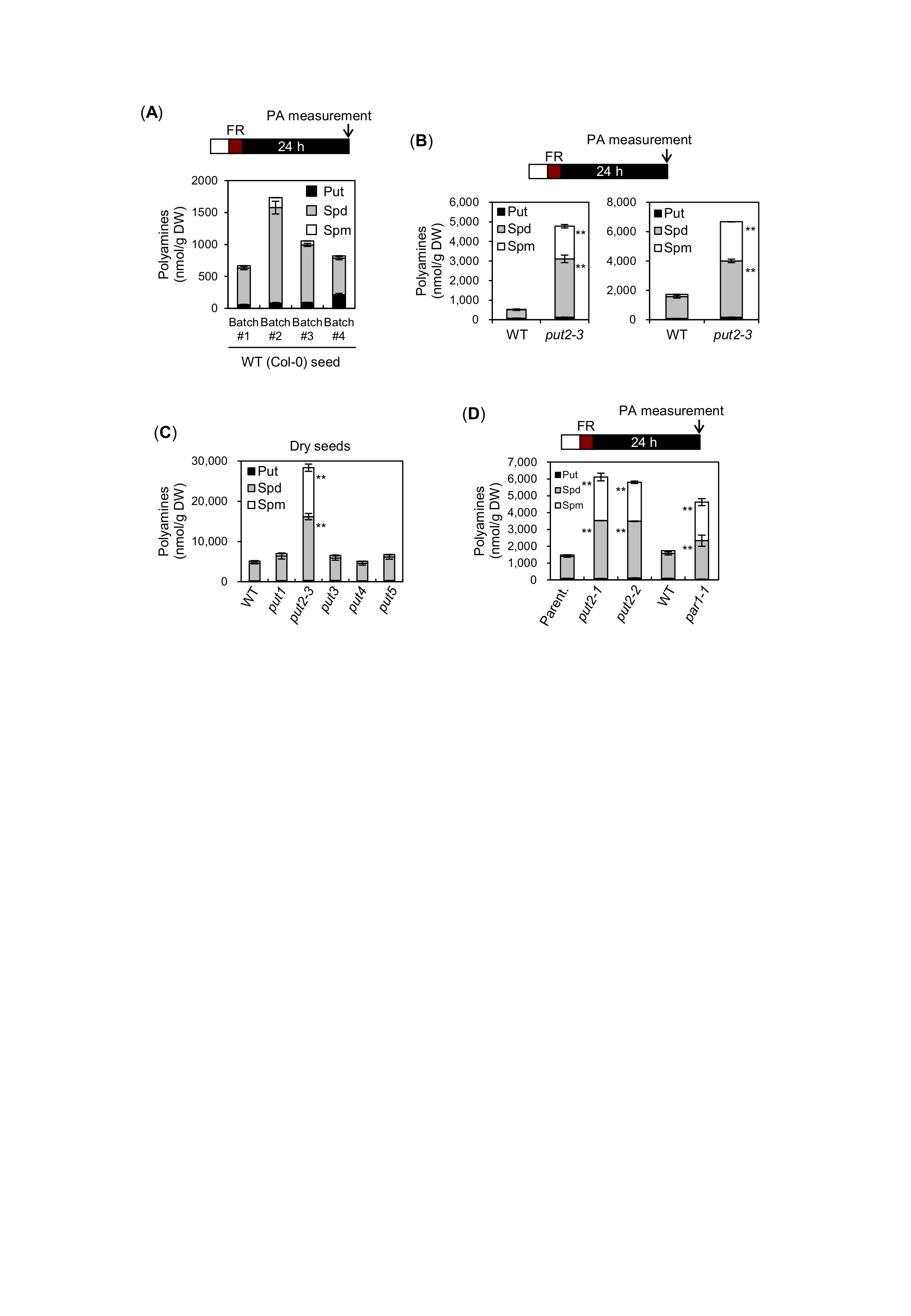

Supplement: S6 Fig — (A) Free PA (Put, Spd and Spm) levels in four different Col-0 (WT) seed batches exposed to a FR assay at the indicated time point. (B) Free PA (Put, Spd and Spm) levels in two different seed batches of WT and put2-3 mutant exposed to a FR assay at the indicated time point. (C) Free PA (Put, Spd and Spm) levels in WT and put1—put5 dry seeds. (D) Free PA (Put, Spd and Spm) levels in Parent., WT and different put2 mutant seeds exposed to a FR assay at the indicated time point. For (A-D) three technical repetitions were used for SD (n = 3). Statistical treatment as in Fig 1B. (TIF) [file pgen.1008292.s006.tif]

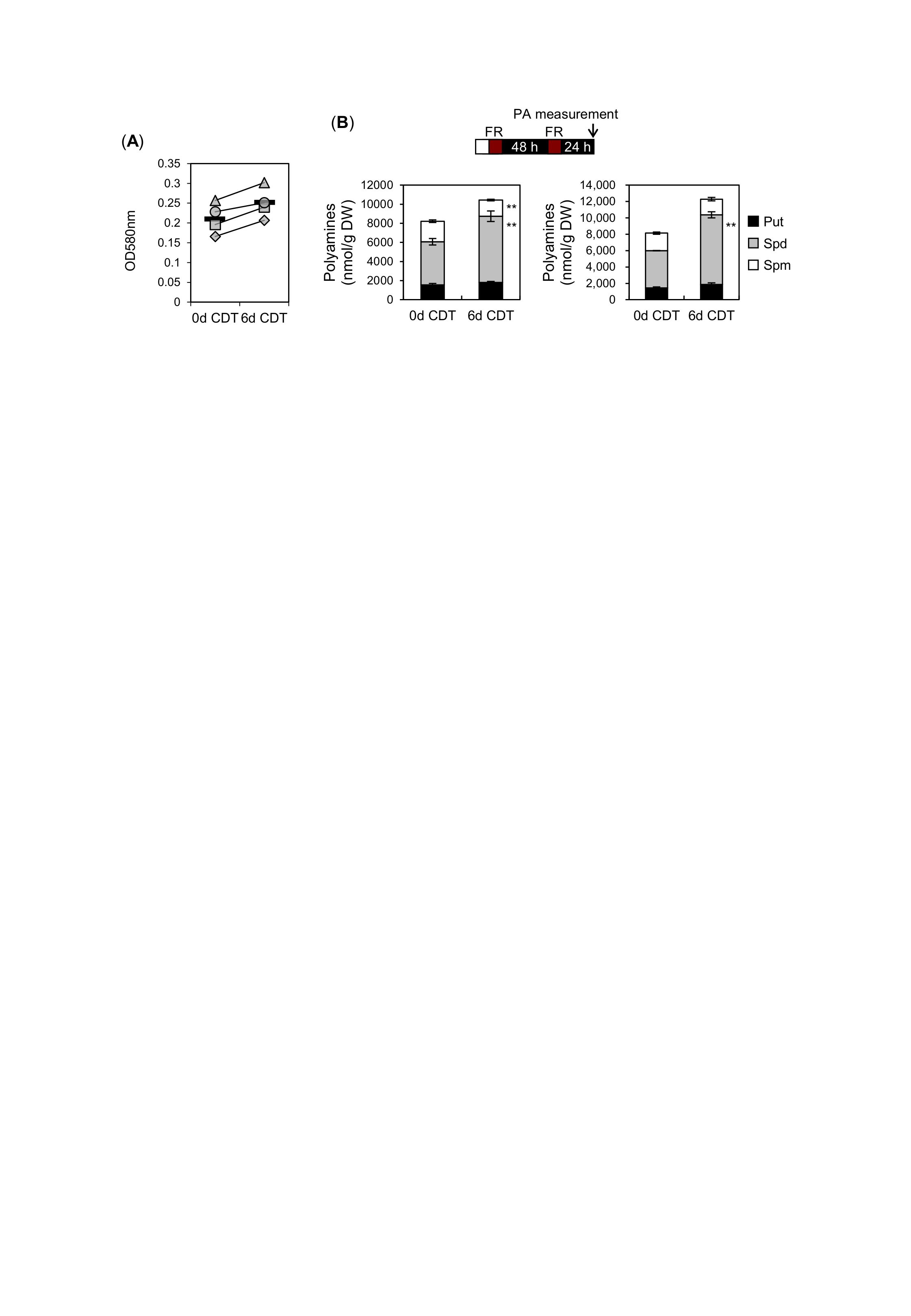

Supplement: S7 Fig — (A) Determination of superoxide O2- levels in CDT-exposed WT dry seeds. Four different seed batches were used for the assay and depicted in the graph. (B) Free PA (Put, Spd and Spm) levels in two different seed batches of CDT-exposed WT seeds. PAs were measured 24 h after the second FR pulse. Three technical repetitions were used for SD (n = 3). Statistical treatment as in Fig 1B. (TIF) [file pgen.1008292.s007.tif]

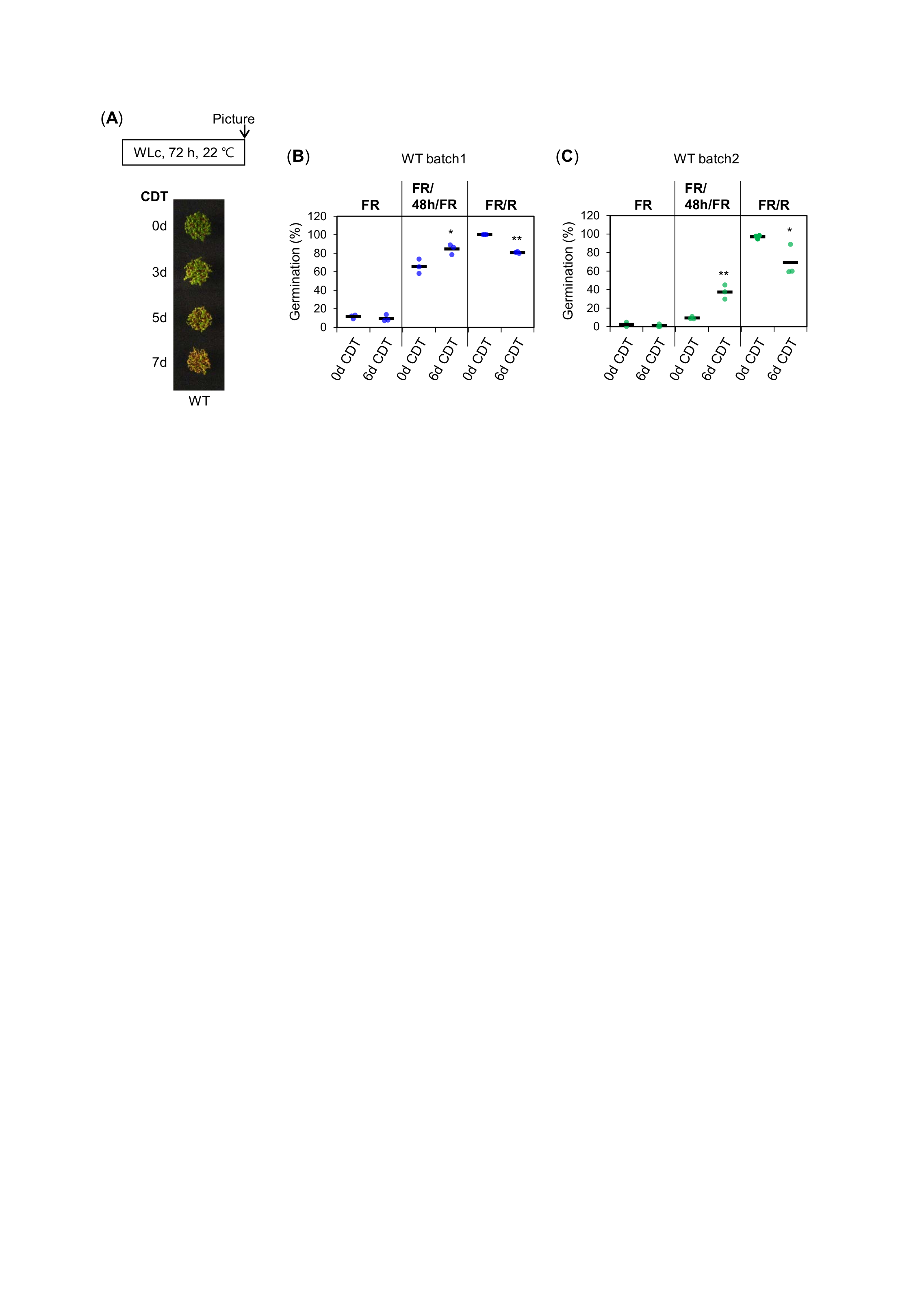

Supplement: S8 Fig — (A) Picture shows different days of CDT-exposed WT seeds grown under constant white light (WLc) for 72 h. (B and C) Germination percentage of two different seed batches of CDT-exposed WT seeds in a FR, FR/48h/FR or FR/R assay. For each repetition, the germination percentage of 50–65 seeds is shown by a blue (B) or green (C) dot. The average germination percentage for the three repetitions is represented by a horizontal black bar. Statistical treatment as in Fig 1B. (TIF) [file pgen.1008292.s008.tif]

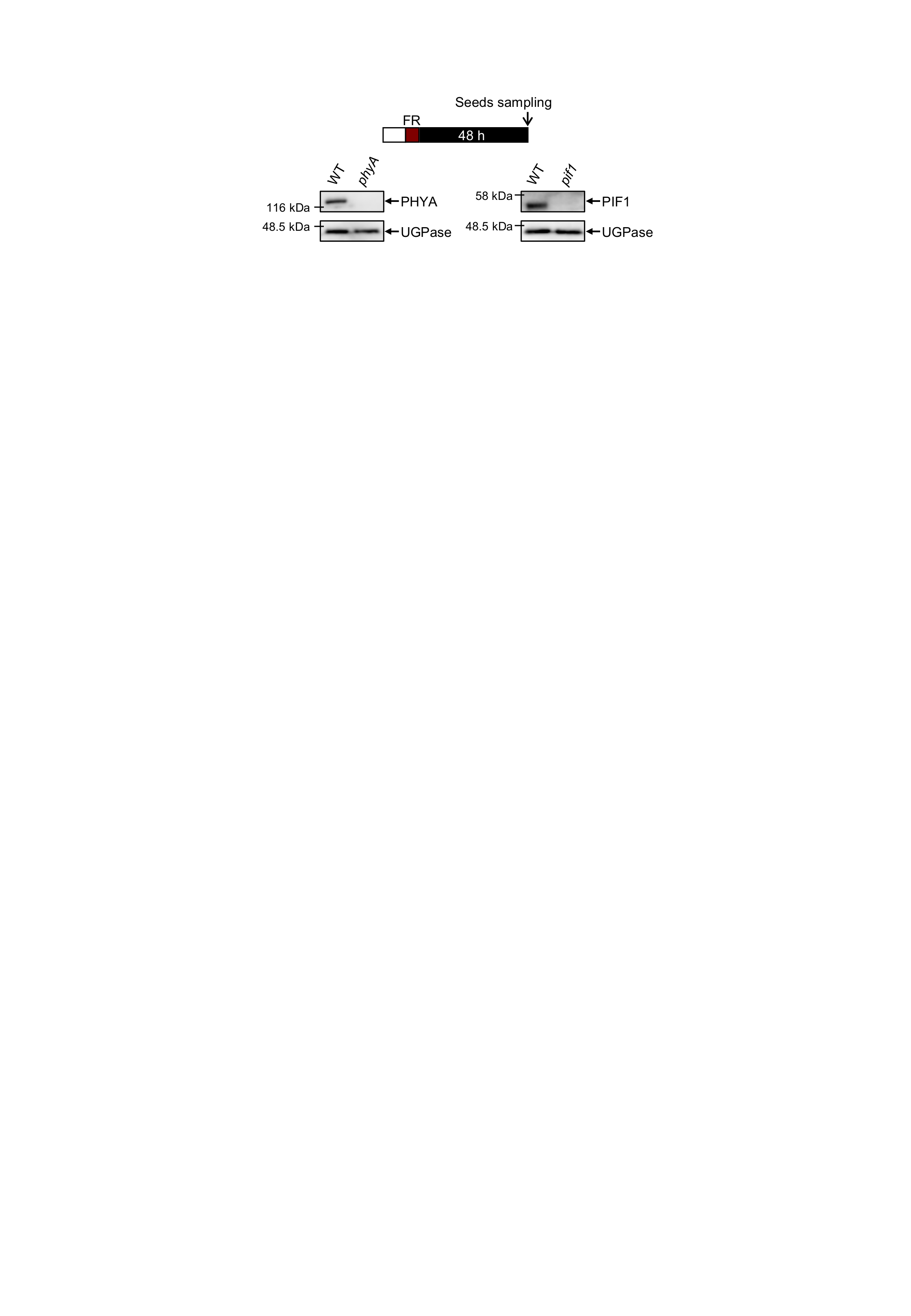

Supplement: S9 Fig — Protein gel blot analysis of phyA protein levels in WT and phyA seeds (left), and PIF1 protein levels in WT and pif1 seeds (right) exposed to a FR assay at the indicated time point after the FR pulse. UGPase protein levels were used as a loading control. (TIF) [file pgen.1008292.s009.tif]

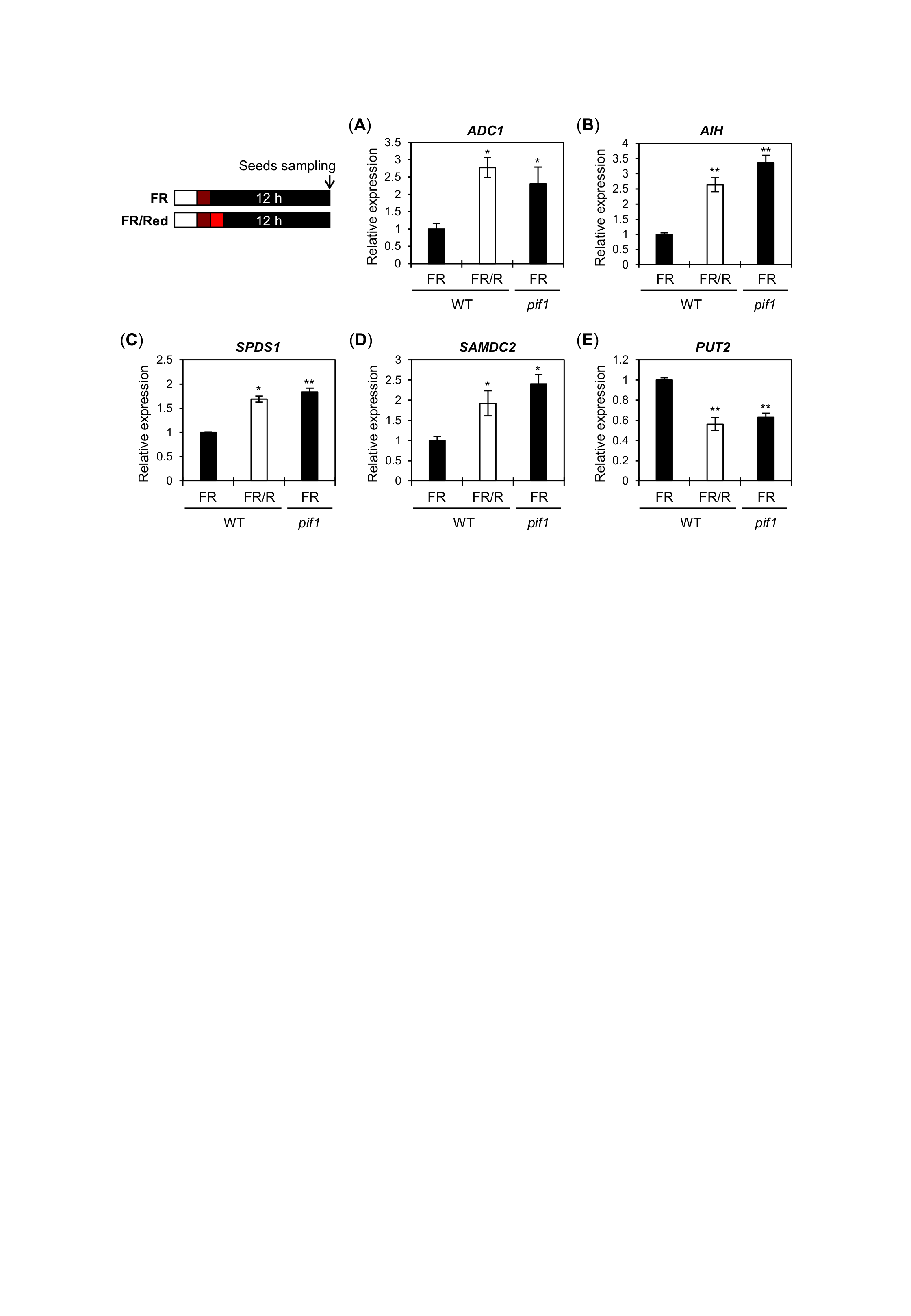

Supplement: S10 Fig — (A-E) Publicly available microarray expression data of PA biosynthesis genes: ARGININE DECARBOXYLASE 1; ADC1 (A), AGMATINE IMINOHYDROLASE; AIH (B), SPERMIDINE SYNTHASE 1; SPDS1 (C), S-ADENOSYLMETHIONINE DECARBOXYLASE 2; SAMDC2 (D), and PUT2 (E) in WT and pif1 seeds exposed to a FR or FR/R assay. Microarray data were provided by Dr. Giltsu Choi. Two technical repetitions were used for SD (n = 2). Statistical treatment as in Fig 1B. (TIF) [file pgen.1008292.s010.tif]

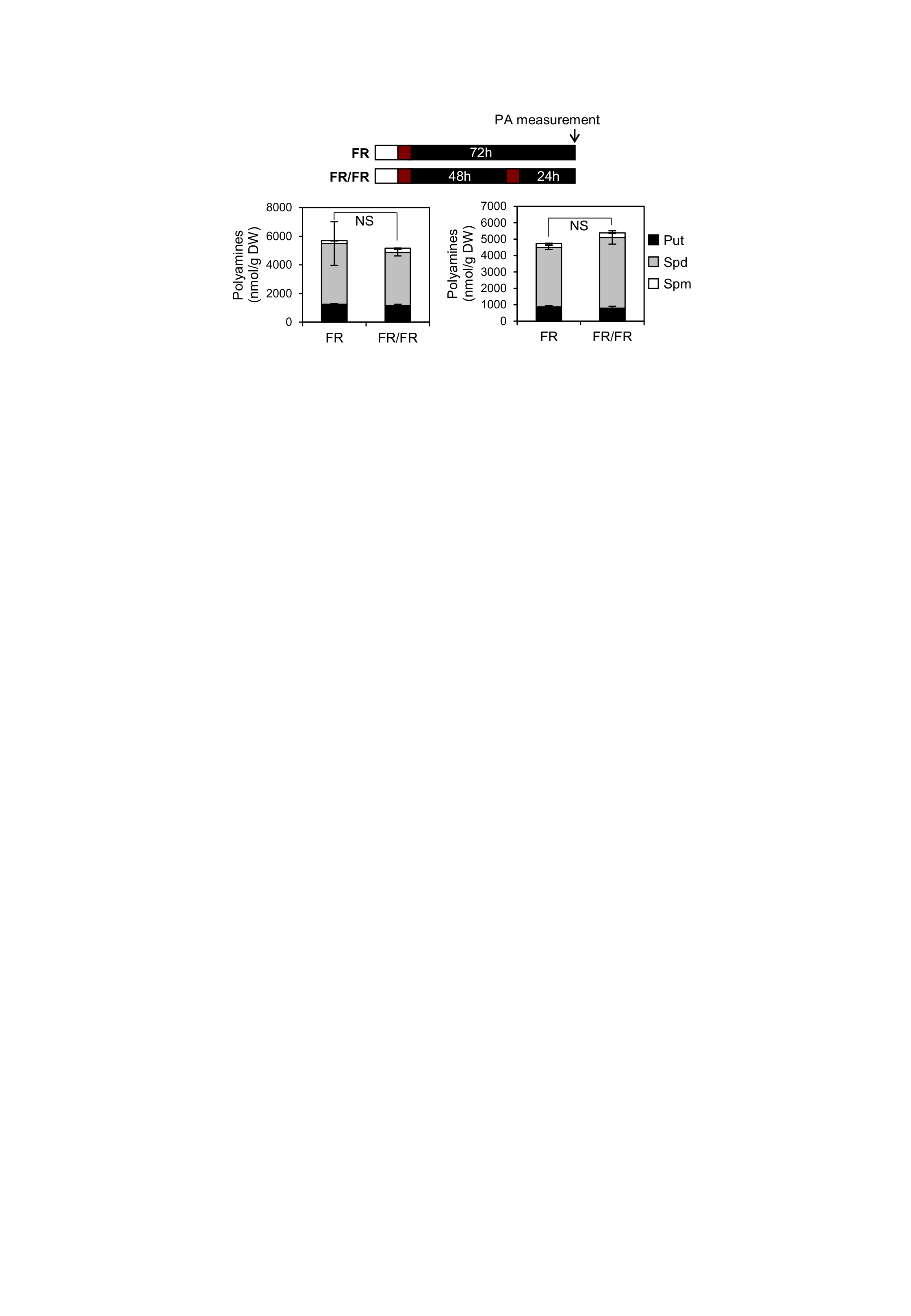

Supplement: S11 Fig — Free PA (Put, Spd and Spm) levels in two different seed batches of WT seeds exposed to a FR or FR/48h/FR assay. PAs were measured 72 h after the first FR pulse or 24 h after the second FR pulse. Three technical repetitions were used for SD (n = 3). Statistical treatment as in Fig 1B. NS: Not Significant. (TIF) [file pgen.1008292.s011.tif]

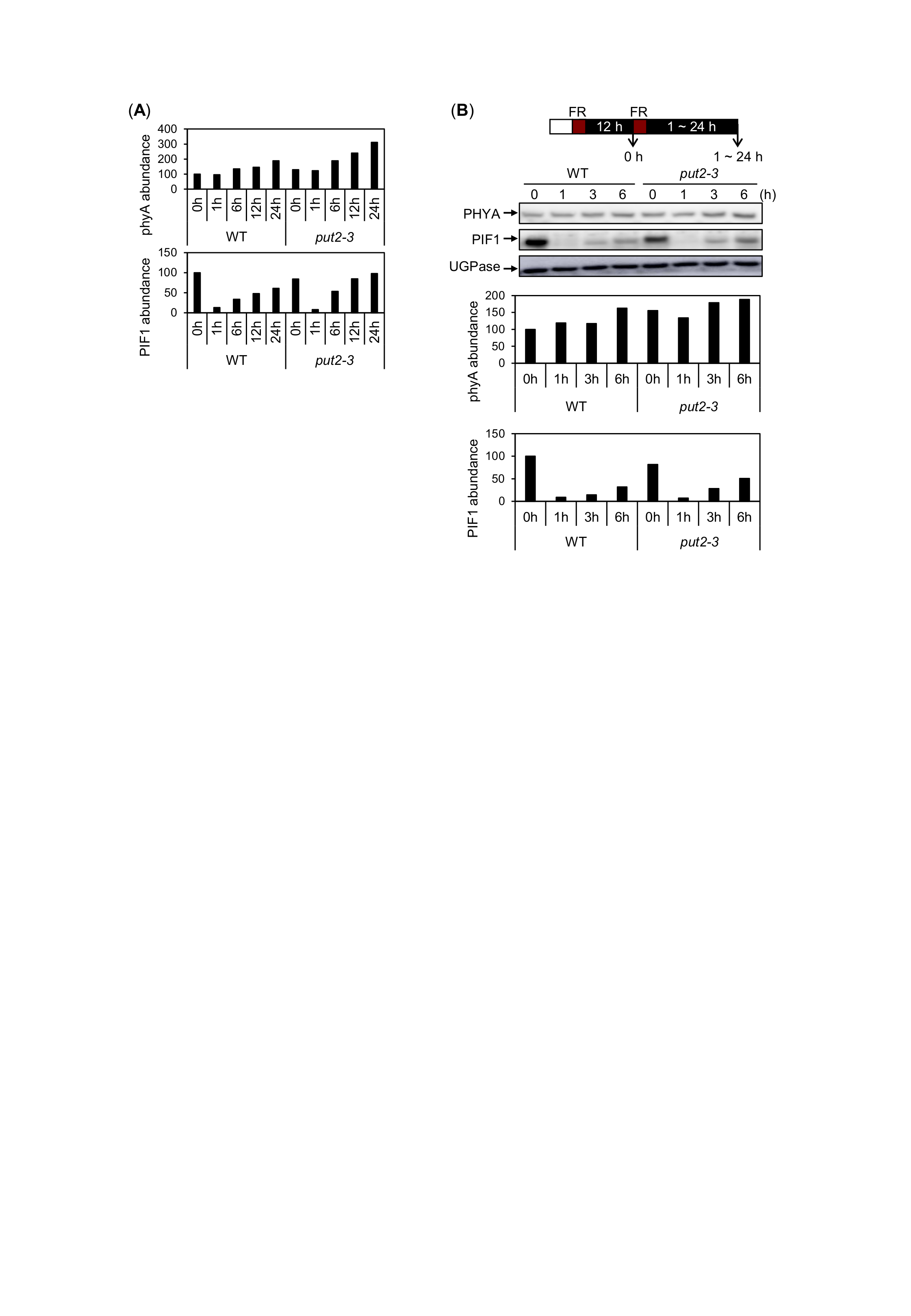

Supplement: S12 Fig — (A) Quantification of Fig 7C. Accumulation of phyA and PIF1 protein levels is shown. (B) Biological replicates of Fig 7C and accumulation of phyA and PIF1 protein levels is shown. (TIF) [file pgen.1008292.s012.tif]

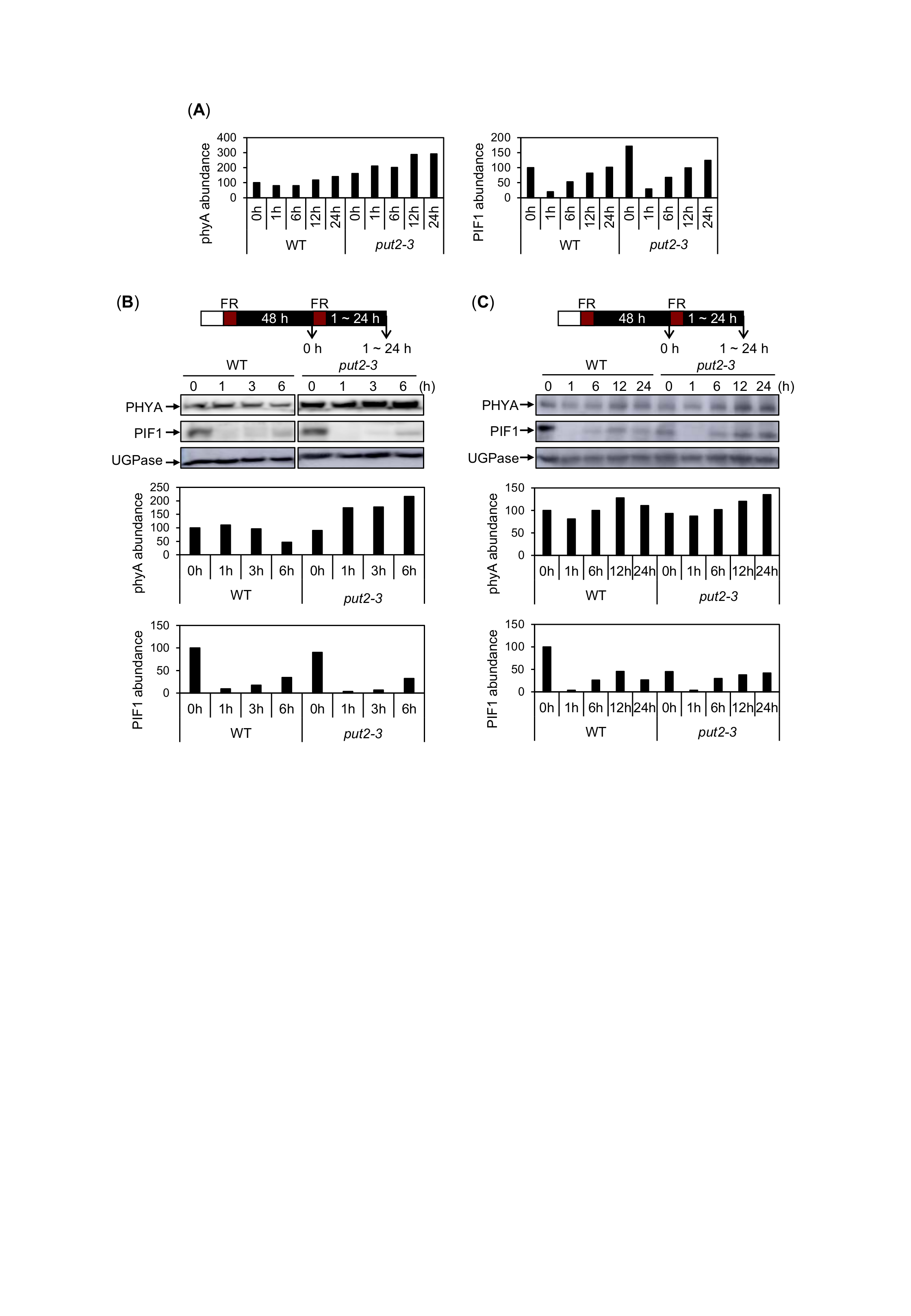

Supplement: S13 Fig — (A) Quantification of Fig 7D. Accumulation of phyA and PIF1 protein levels is shown. (B and C) Biological replicates of Fig 7D and accumulation of phyA and PIF1 protein levels is shown. (TIF) [file pgen.1008292.s013.tif]

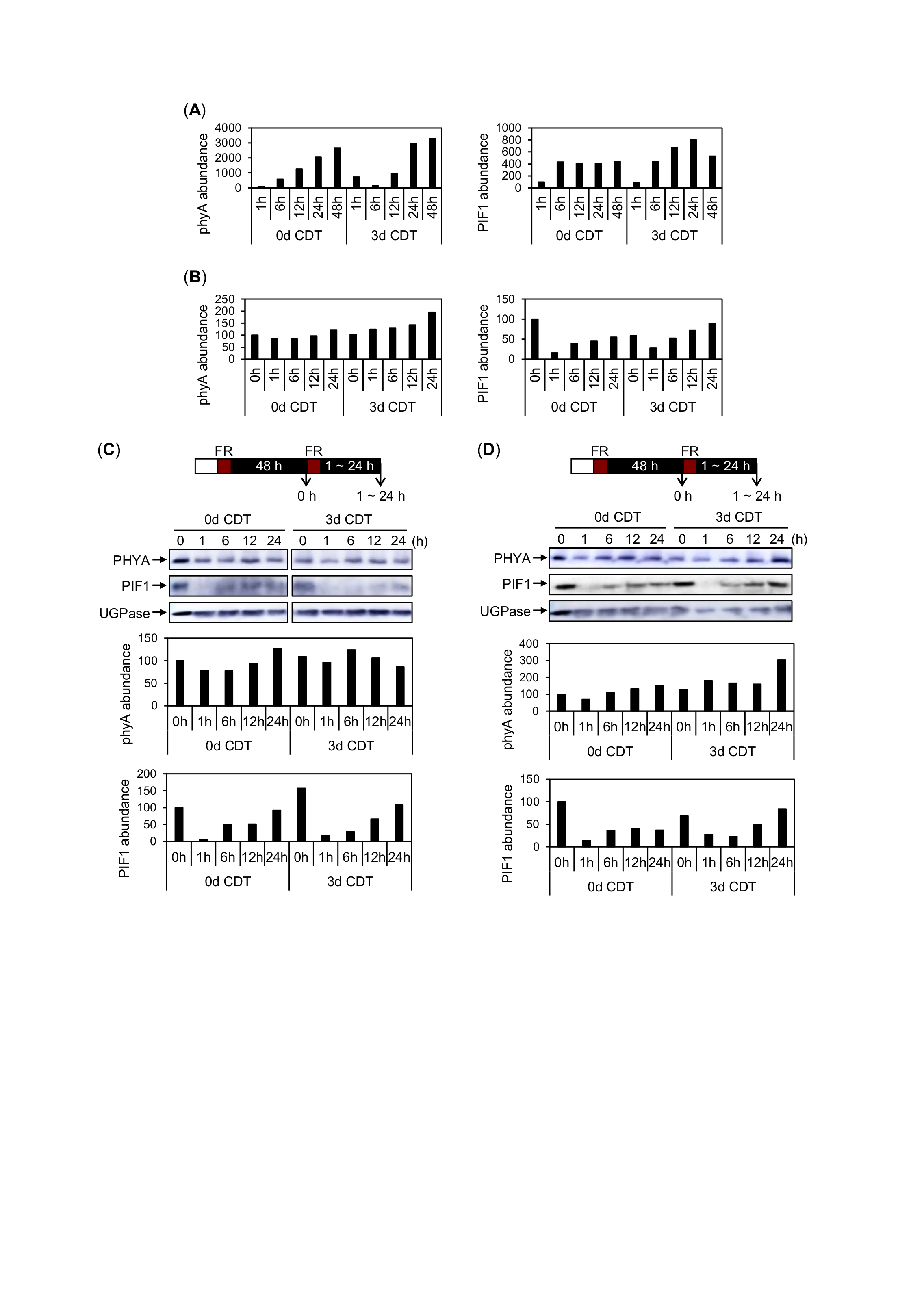

Supplement: S14 Fig — (A and B) Quantification of Fig 7E upper (A) and bottom (B). Accumulation of phyA and PIF1 protein levels is shown. (C and D) Biological replicates of Fig 7E and accumulation of phyA and PIF1 protein levels is shown. (TIF) [file pgen.1008292.s014.tif]

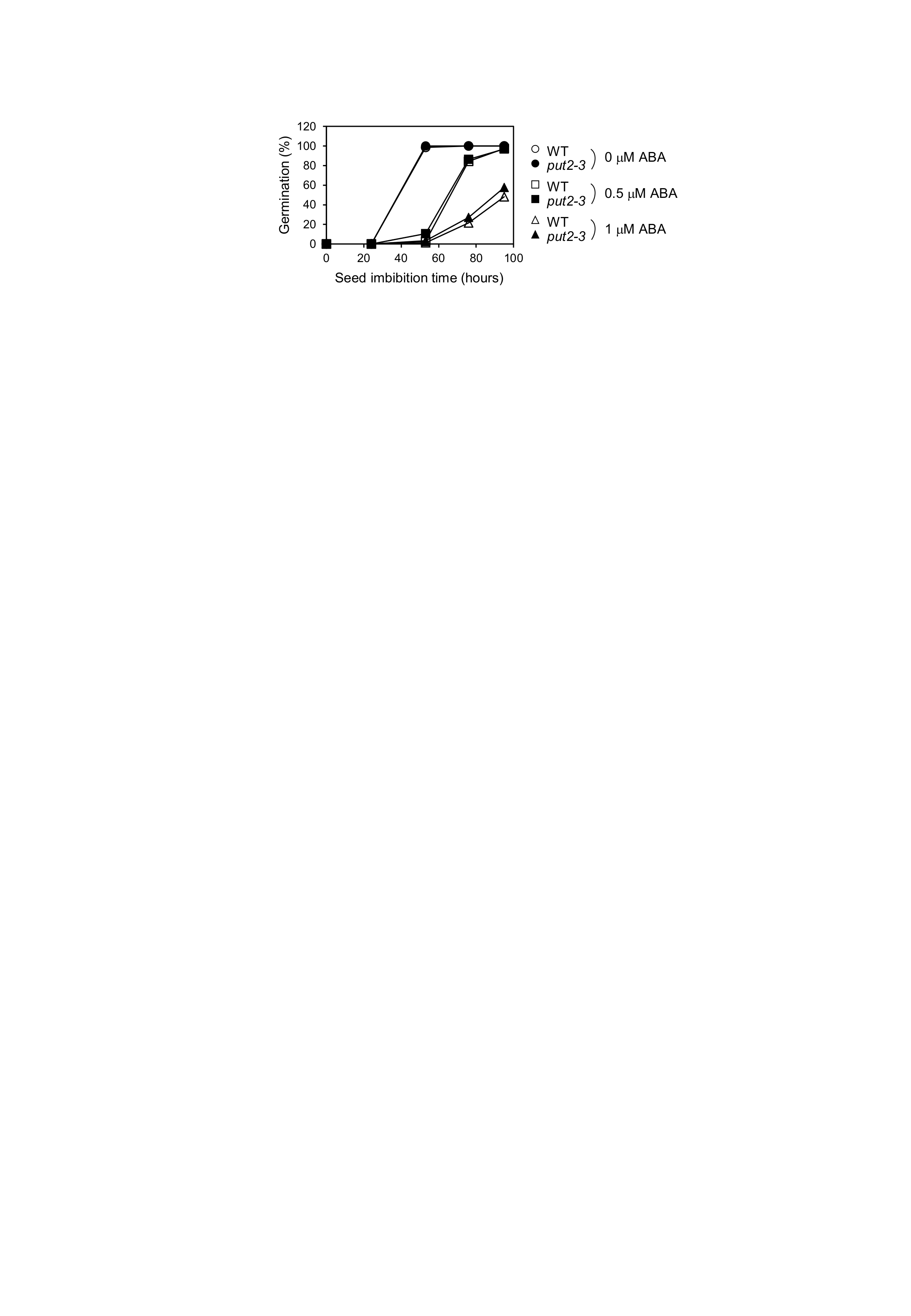

Supplement: S15 Fig — Graph shows germination percentage of WT and put2-3 mutant seeds in absence or presence of different concentrations of ABA. Seeds were grown under constant white light and germination percentage (radicle protrusion) was scored at the indicated time points. (TIF) [file pgen.1008292.s015.tif]
